# Valence-Change MnO_2_-Coated Arsenene Nanosheets as a Pin1 Inhibitor for Hepatocellular Carcinoma Treatment

**DOI:** 10.1021/jacs.4c05162

**Published:** 2024-07-25

**Authors:** Jingguo Wang, Siping Liang, Dongdong Zhu, Xiaocao Ma, Qin Peng, Guanzhao Wang, Yuting Wang, Tiantian Chen, Minhao Wu, Tony Y. Hu, Yuanqing Zhang

**Affiliations:** † Zhongshan School of Medicine, Sun Yat-Sen University, Guangdong 510080, China; ‡ School of Pharmaceutical Sciences, Sun Yat-Sen University, Guangdong 510006, China; § Center of Cellular and Molecular Diagnosis, Tulane University School of Medicine, New Orleans, Louisiana 70112, United States

## Abstract

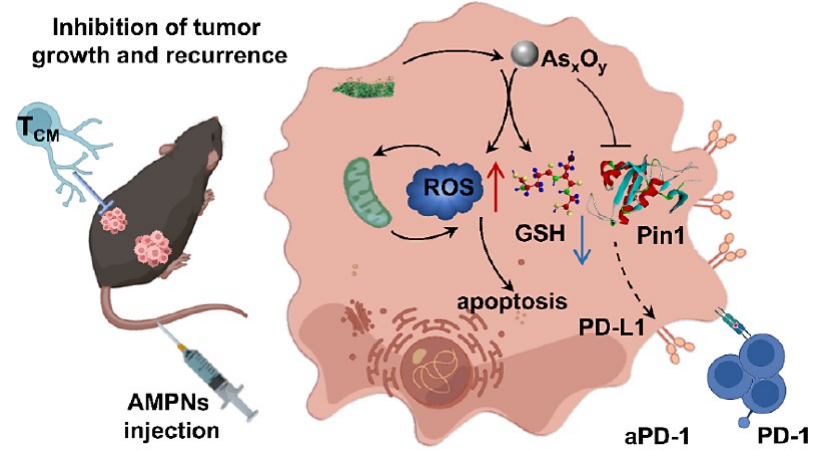

The heterogeneity of hepatocellular carcinoma (HCC) can prevent effective treatment, emphasizing the need for more effective therapies. Herein, we employed arsenene nanosheets coated with manganese dioxide and polyethylene glycol (AMPNs) for the degradation of Pin1, which is universally overexpressed in HCC. By employing an “AND gate”, AMPNs exhibited responsiveness toward excessive glutathione and hydrogen peroxide within the tumor microenvironment, thereby selectively releasing As_*x*_O_*y*_ to mitigate potential side effects of As_2_O_3_. Notably, AMPNs induced the suppressing Pin1 expression while simultaneously upregulation PD-L1, thereby eliciting a robust antitumor immune response and enhancing the efficacy of anti-PD-1/anti-PD-L1 therapy. The combination of AMPNs and anti-PD-1 synergistically enhanced tumor suppression and effectively induced long-lasting immune memory. This approach did not reveal As_2_O_3_-associated toxicity, indicating that arsenene-based nanotherapeutic could be employed to amplify the response rate of anti-PD-1/anti-PD-L1 therapy to improve the clinical outcomes of HCC patients and potentially other solid tumors (e.g., breast cancer) that are refractory to anti-PD-1/anti-PD-L1 therapy.

## Introduction

Hepatocellular carcinoma (HCC) is the most common type of primary liver cancer and stands as one of the most frequently occurring malignancies worldwide.^1,2^ The median overall survival of HCC patients hovers around meager 11–14 months, which is attributed to the heterogeneity of HCC mutations and phenotypes.^3−5^ Despite the promising prospects of immunotherapeutic approaches, including immune checkpoint inhibitor (ICI) treatment, low overall response rates due to interindividual variability remain a persistent challenge.^6−9^ Evidence suggests this is related to altered expression in HCC tumors (e.g., low PD-L1 expression) and their tumor immune microenvironment (TIME).^8−12^ Thus, it is crucial to address mechanisms that regulate HCC pathogenesis and TIME phenotypes before ICI therapies.

The prolyl isomerase Pin1, which primarily localizes to the nucleus, contains a substrate recognition and catalysis domain,^13−16^ and numerous studies have revealed that overactive or overexpressed Pin1 can elicit adverse HCC clinical outcomes.^17−19^ Pin1 induces PD-L1 endocytosis and lysosomal degradation pathways to attenuate the efficacy of ICI.^20,21^ Pin1 inhibitors, including walnut ketone and As_2_O_3_, can promote Pin1 degradation,^20−23^ which could increase ICI efficacy, but exhibit off-target effects and poor in vivo stability that limit their potential for further development as therapeutics.^22,24,25^ There is thus an urgent need to develop selective, effective, and safe Pin1 inhibitors to combat HCC.

As_2_O_3_ is an ancient drug and has been used to treat highly lethal acute promyelocytic leukemia, where it can achieve a >90% rate of complete remission.^26−29^ This success has inspired researchers to evaluate the potential to expand its application range, but the low bioavailability and high systemic toxicity of As_2_O_3_ limit its therapeutic efficacy when it is used to treat solid tumors.^30−34^ Metallic arsenic (As^0^) has experimentally demonstrated a layered structure, exhibiting a buckled honeycomb structure similar to its same family of phosphorus and antimony.^35−39^ Moreover, it demonstrates exceptional optical properties and a remarkable ability to induce apoptosis in tumor cells. More significantly, in comparison to As_2_O_3_, mono elemental arsenene exhibits remarkable stability and biocompatibility.^37,38^ Based on these findings, PEGylated arsenide nanodots synthesized by Liu et al. exhibit selective cytotoxicity toward tumor cells by inducing apoptosis through mitochondrial damage and reactive oxygen species (ROS) burst while demonstrating no significant side effects on normal cells.^38^ The difference in cytotoxicity may be attributed to the increased oxidative stress in tumor cells, characterized by a high concentration of hydrogen peroxide (H_2_O_2_) in the tumor microenvironment (TME).^38−40^ This heightened oxidative environment is believed to facilitate the conversion of As^0^ into As_2_O_3_/As_2_O_5_ (As_*x*_O_*y*_), thus inducing apoptosis in cancer cells. These findings indicate that delivering arsenene specifically to tumors could be a safe and effective antitumor strategy.

**Figure 1:**
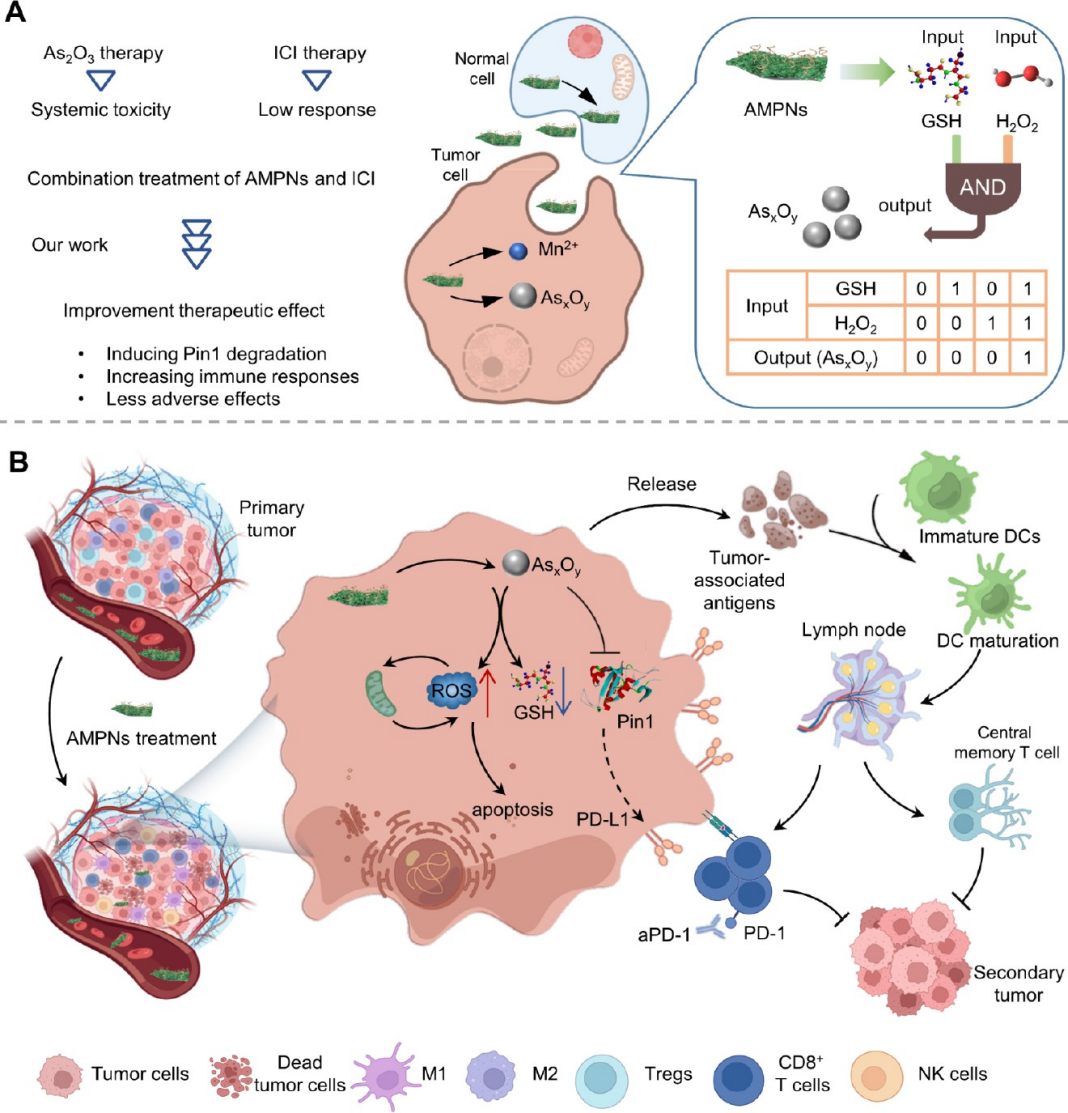
AMPNs “AND gate” and regulation of tumor and TME effects. (A) AMPNs “AND gate” regulation of As_*x*_O_*y*_ release. (B) APMNs inhibit Pin1 expression to induce ROS production, mitochondrial injury, and apoptosis and to enhance responses to ICI therapy.

Herein, we describe the synthesis, characteristics and therapeutic activity of As/MnO_2_–PEG nanosheets (AMPNs) where As_*x*_O_*y*_ release is regulated by dual exposure to high levels of glutathione (GSH) and H_2_O_2_, as occurs in the TME, to prevent toxicity due to As_*x*_O_*y*_ leakage in other tissues under normal physiologic conditions (Figure 1A). In this approach, GSH in the TME reacts with the external MnO_2_ layer of the AMPNs to allow TME H_2_O_2_ to liberate As_*x*_O_*y*_ that can have an array of therapeutic effects. We now demonstrate that AMPNs stimulate more ROS production, mitochondrial depolarization and injury, and apoptosis than arsenene nanosheets (ANs) dose-equivalent and exhibit greater effects to decrease Pin1 and increase PD-L1 expression and reduce tumor cell growth, migration, and invasion activity (Figure 1B). We also report that by inhibition of Pin1, AMPNs plus anti-PD-1/anti-PD-L1 treatment produce superior tumor inhibition and clearance in correspondence with tumor suppressive effects induced in the TIME, and greater suppression of tumor development when these mice are rechallenged with the same cancer cells post-treatment. Notably, the therapeutic effect was similarly confirmed in a mouse breast cancer model overexpressing Pin1. These results strongly imply that arsenene-based nanomedicine could augment the efficacy of ICI therapy to improve patient outcomes by inhibiting Pin1.

## Results

### HCC Pin1 Expression and its TIME Implications

**Figure 2:**
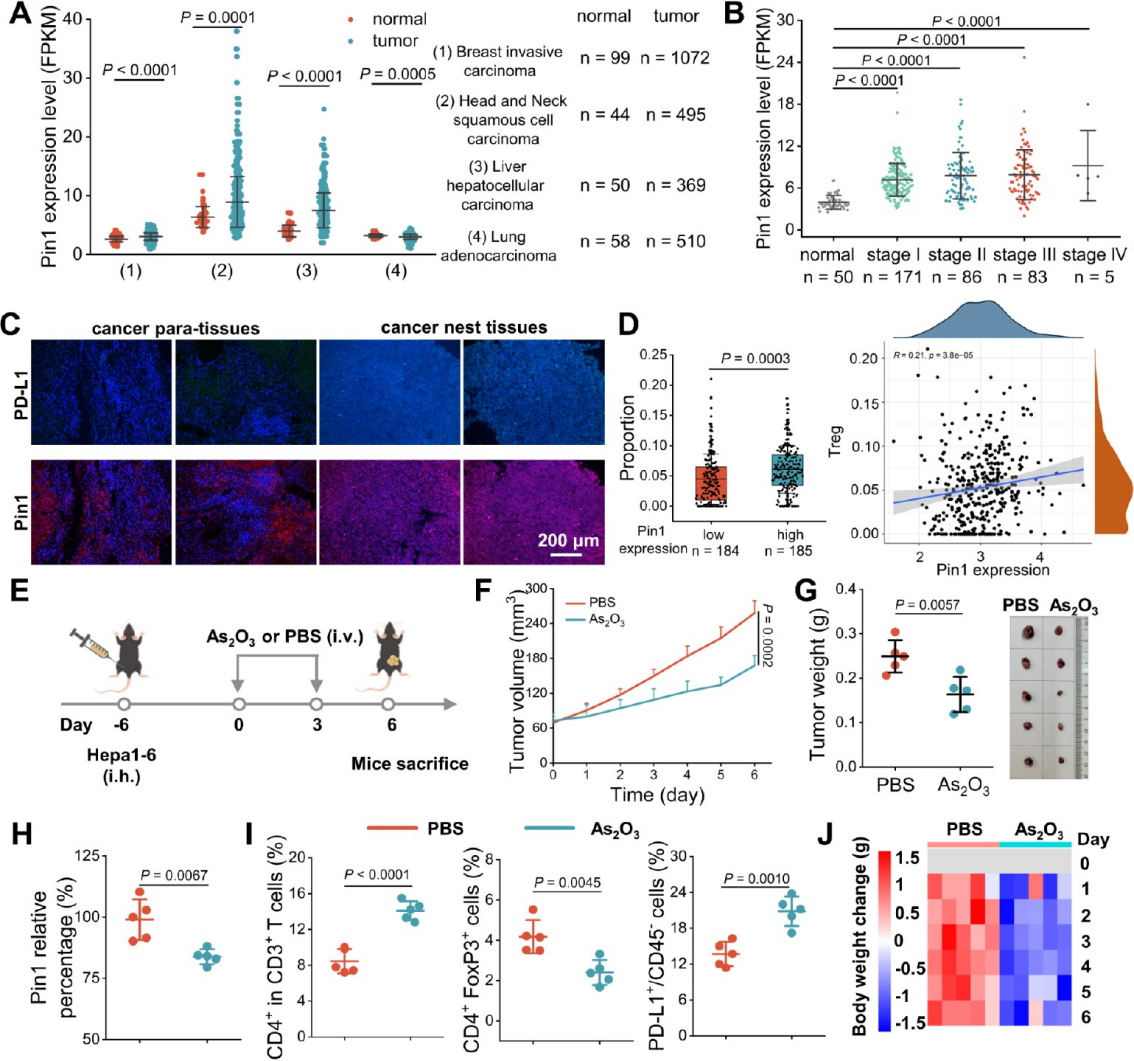
Pin1 expression in HCC and its implications for HCC TIME. (A) Pin1 expression in tumor and normal tissues. (B) Pin1 expression in normal liver tissue vs different HCC stages. (C) Immunofluorescence staining of HCC paratumor and cancer nest tissues. (PD-L1: green; Pin1: red). Scale bars, 200 μm. (D) Spearman correlations between Pin1 expression and Treg cells. (E) Schematic of the mouse tumor growth model design. (F) Six-day tumor growth curves. (G) Excised tumor weights and images at day 6. (H) Relative percentage of Pin1 expression in tumor tissue in PBS- or As_2_O_3_-treated mice. (I) CD4^+^ T cells, Treg, and PD-L1^+^ cell frequencies in tumors of expression on tumor cells. (J) Mouse body weight changes during treatment.

Since Pin1 can upregulate more than 60 oncoproteins and downregulate over 30 tumor suppressor proteins,^14,19,20^ we analyzed Pin1 in different cancer types by a bioinformatic search of the cancer genome atlas (TCGA) database. This analysis revealed significant Pin1 overexpression in various cancers versus normal tissue, including breast cancer, pancreatic cancer, and HCC (Figures 2A and S1). Notably, Pin1 expression was elevated at all HCC stages (Figure 2B), and robust Pin1 expression was detected in both paracancerous tissue and cancer nests (Figures 2C and S2). However, PD-L1 expression in paracancerous or cancer nests was hardly detectable, likely due to Pin1 overexpression promoting the degradation of PD-L1.^20^ CD4^+^ and CD8^+^ T cell, macrophage, and natural killer (NK) cell infiltration were negatively correlated, and Treg infiltration was negatively correlated with Pin1 expression (Figures 2D and S3). Thus, it appears that Pin1 inhibition may not only affect ICI efficacy but also reshape the TIME.

To evaluate this hypothesis, we treated mice carrying Hepa1-6 tumors with serial intravenous doses of As_2_O_3_, a customary Pin1 inhibitor,^22^ to evaluate the effect of Pin1 inhibition on tumor growth and the TIME (Figure 2E). As_2_O_3_ treatment substantially repressed tumor growth after treatment initiation (Figure 2F), final tumor weight at necropsy (Figure 2G), and Pin1 expression (82.2–96.2% PBS control) (Figure 2H). Notably, flow cytometry (FC) analysis of tumor tissue also revealed marked CD4^+^ and CD8^+^ T cell increases and Treg decreases (Figures 2I and S4) in the As_2_O_3_-treated group as well as an increase in the number PD-L1 expressing nonlymphoid tumor cells. However, while As_2_O_3_ treatment appeared to promote the development of tumor-suppressive TIME, 80% of the As_2_O_3_-treated mice lost weight. This weight loss occurred within 1 day of treatment initiation and persisted through treatment, with some mice losing >3.3 g of body weight (Figure 2J). Furthermore, hematoxylin-eosin (H&E) staining of the organs of these mice revealed significant liver and kidney injury (Figure S5).

### AMPNs Production and Characterization

**Figure 3:**
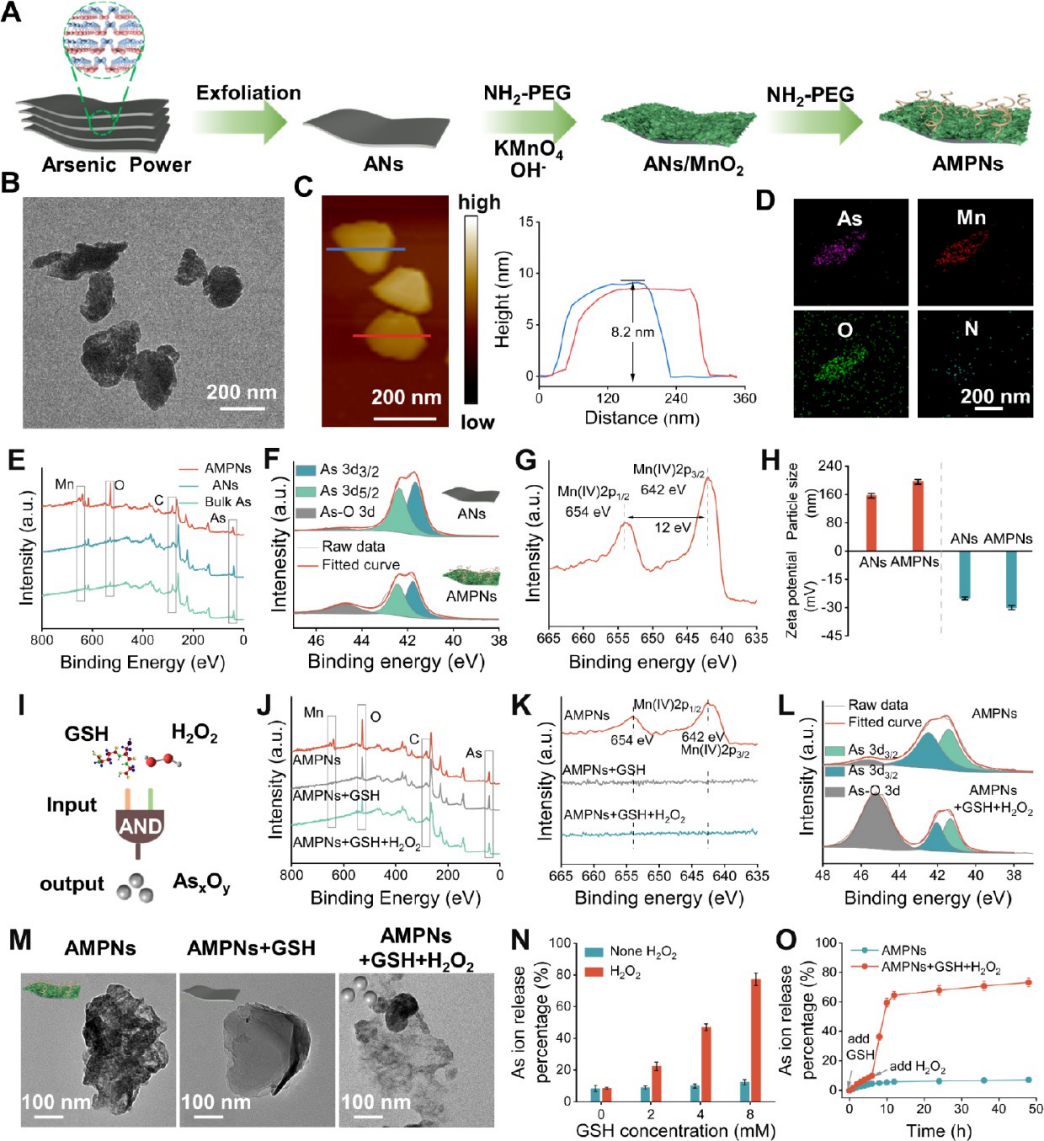
Characterization of AMPNs “AND” logic gate-controlled As_*x*_O_*y*_ release. (A) Schematic of the AMPNs synthesis process. (B–D) TEM (B), AFM (C), and EDS (D) mapping analysis of AMPNs. Scale bars, 200 nm. (E–G) XPS survey spectra (E), As 3d (F), and Mn 2p (G) spectra of bulk As, ANs, and AMPNs. (H) Particle size and zeta potential of ANs and AMPNs. (I) Schematic of the GSH and H_2_O_2_ “AND gate” of AMPNs. (J–L) XPS (J), Mn 2p (K), and As 3d (L) spectra of AMPNs incubated with GSH and H_2_O_2_. (M) TEM images of AMPNs after incubation with or without GSH and H_2_O_2_. Scale bars: 100 nm. (N) As ion release at different GSH concentrations with or without H_2_O_2_ exposure. (O) Cumulative release profiles of As ions from AMPNs with and without GSH and H_2_O_2_ exposure.

Since As_2_O_3_ treatment had beneficial tumor and TIME effects but significant toxicity, we next analyzed whether modified ANs could provide similar Pin1 inhibition and antitumor effects as As_2_O_3_ but with reduced toxicity. ANs generated by liquid-phase exfoliation^37^ were coated with MnO_2_ and NH_2_–PEG to obtain AMPNs (Figure 3A).

As the first step in this process, arsenene powder was submerged in deionized water containing NH_2_–PEG and then sonicated to produce ANs. Scanning electron microscope (SEM) images of the bulk arsenene powder and transmission electron microscope (TEM), high-resolution transmission electron microscope (HRTEM), and atomic force microscopy (AFM) images of the resulting ANs revealed plane size, lattice, and thickness spacing values of approximately 180, 0.26, and 2.6 nm (Figure S6A–C), indicating successful exfoliation of the ANs generated in our approach. X-ray diffraction (XRD) peaks matched those of As (JCPDS No. 00-001-0760), while characteristic ANs peaks observed at 202.8 and 258.1 cm^–1^ were attributed to Eg (in-plane vibration) and A1g (out-of-plane vibration) (Figure S6D,E).^33,37^ Energy-dispersive X-ray spectrometer (EDS) data detected an As content >95%, indicating that a negligible amount of As was oxidized in the final AN samples (Figure S6F). These results demonstrated the successful synthesis of a nonoxidized AN material used for the subsequent AMPN synthesis process.

These ANs were next reduced with KMnO_4_ to produce MnO_2_-modified ANs that were then conjugated with NH_2_–PEG to obtain AMPNs.^41,42^ TEM and AFM images of these AMPNs revealed that the planar size and thickness values of the initial ANs increased from approximately 180 to 210 nm and from 2.6 to 8.2 nm (Figure 3B,C). EDS mapping and X-ray photoelectron spectroscopy (XPS) revealed the incorporation and colocalization of As, Mn, O, and N onto these AMPNs (Figure 3D,E). The quantitative analysis of XPS spectra revealed ratios of As–O 3d to As 3d peaks that were determined as 0.03:1 and 0.09:1, respectively, indicating a moderate oxidation effect in the preparation of AMPNs (Figure 3F). Furthermore, the presence of MnO_2_ in AMPNs was confirmed by the appearance of XPS peaks at energies corresponding to 642 and 654 eV (Figure 3G). For Fourier transform infrared (FT-IR) spectra, compared with ANs/MnO_2_, the appearance of absorption bands at about 1100 and 2900 cm^–1^ belongs to CH_2_CH_2_O and CH_2_ vibrations of NH_2_–PEG (Figure S7), and changes in UV–Vis absorption spectra, particle size, and zeta potential further supportedsuccessful AMPN preparation (Figures 3H and S8).

### Dual Regulation of As_*x*_O_*y*_ Release by AMPNs

AMPNs are expected to produce negligible As_*x*_O_*y*_ before entering the TME based on their physicochemical properties,^43^ as simultaneous exposure to high GSH and H_2_O_2_ levels preferentially found in many TME is required to promote arsenene conversion to As_*x*_O_*y*_ conversion through a two-stage “AND gate” process. The AMPNs were dispersed in various solvents, and no significant changes in particle size were observed within 7 days, indicating the robust stability of AMPNs. High-concentration GSH exposure is required for efficient degradation of surface MnO_2_ to permit local H_2_O_2_ to subsequently oxidize the internal ANs core and liberate free As_*x*_O_*y*_ (Figure 3I). XPS analysis of AMPNs incubated with or without GSH or GSH + H_2_O_2_ revealed a decrease in the Mn and O peaks in the GSH-treated samples, consistent with degradation of the MnO_2_ coating (Figure 3J,K). However, the decrease of the As peak was only observed in GSH + H_2_O_2_-treated samples, as confirmed by the increase of the peak area ratio of As–O to As 3d_3/2_ and As 3d_5/2_ from 0.08:1 to 1.78:1 (Figure 3L). TEM images of these samples revealed that GSH treatment removed the AMPNs MnO_2_ surface coating, while GSH and H_2_O_2_ co-incubation was required to degrade ANs structural integrity (Figure 3M). To quantify As_*x*_O_*y*_ release rates for AMPNs exposed to GSH or GSH + H_2_O_2_, AMPNs (500 μg As) loaded in dialysis tubes (7000 Da exclusion) were incubated in increasing GSH concentrations in the presence and absence of H_2_O_2_ for 48 h, and free As_*x*_O_*y*_ was measured by inductively coupled plasma mass spectrometry (ICP-MS). This analysis detected marked As_*x*_O_*y*_ release only in the presence of H_2_O_2_, and this release was GSH-dependent, as As_*x*_O_*y*_ was not observed without GSH and increased with GSH concentration (Figure 3N). We next analyzed AMPNs stability over extended incubation in PBS, or GSH + H_2_O_2_ concentrations intended to mimic those encountered in TME. GSH + H_2_O_2_-treated AMPNs were first incubated for 6 h in GSH and then incubated for an additional 42 h after the addition of H_2_O_2_. This analysis observed a slow and linear As_*x*_O_*y*_ release rate during the 6 h GSH incubation (7.1% release) followed by a rapid release that tended to plateau within 6 h of H_2_O_2_ addition (73.2% release), with half of the As_*x*_O_*y*_ release detected within 2 h of H_2_O_2_ addition (Figure 3O). Considering the potential damage caused by the MnO_2_ coating in an acidic tumor microenvironment, we further investigated the impact of acidity on both the particle size and the release of arsenic ions from AMPNs. Compared to their stability in a neutral environment, AMPNs exhibited time-dependent degradation behavior in an acidic solution, with the degradation rate positively correlated with the level of acidity (Figure S10A). Similarly, the synergistic effect of an acidic environment and hydrogen peroxide resulted in a significant increase in the cumulative release of arsenic ions over 48 h, escalating from 6.4% to 77.9% (Figure S10B). These results suggest that efficient As_*x*_O_*y*_ release rates should be limited to the TME to promote tumor bioavailability and reduce toxicity from systemic As_*x*_O_*y*_ release, as this should slow to reduce exposure doses and the likelihood of adverse events.

### Selective Killing of AMPNs

Human cell lines used as models of endothelial cells (HUVEC), kidney cells (HEK293), and murine hepatocyte cell lines (AML12) were incubated with different AMPNs concentrations and analyzed. Compared to As_2_O_3_, the cell viability detected and live–dead staining were indicative of the excellent biocompatibility of AMPNs (Figures S11 and S12). Notably, HUVEC and AML12 viability treated with both GSH and H_2_O_2_ and AMPNs were significantly lower than cells incubated with AMPNs alone, which was attributed to the release of As_*x*_O_*y*_ (Figure S13). Hepa1-6 cells incubated with Cy5-labeled AMPNs or ANs revealed that these particles accumulated around the nucleus (Figure S14A,B), and this finding was corroborated by an FC analysis (Figure S14C), suggesting that AMPNs have the ability to internalize cells. AMPN uptake was also markedly higher than observed for ANs under identical experimental conditions, potentially due to the greater stability conferred by the MnO_2_ coating of AMPNs. Notably, confocal images and FC analysis showed Hepa1-6 uptake more for AMPNs than for AML12 (Figure S15), which might be attributed to the dysregulation of the endocytosis pathway in cancer cells.^44^ Moreover, Hoechst and Lyso-Tracker Green staining showed that the red fluorescence of AMPNs merged well with the green fluorescence of lysosomes in the first 2 h and escaped into the cytoplasm over time (Figure S16). The findings suggested an efficient intracellular endocytosis of AMPNs and lysosomal escape.

**Figure 4:**
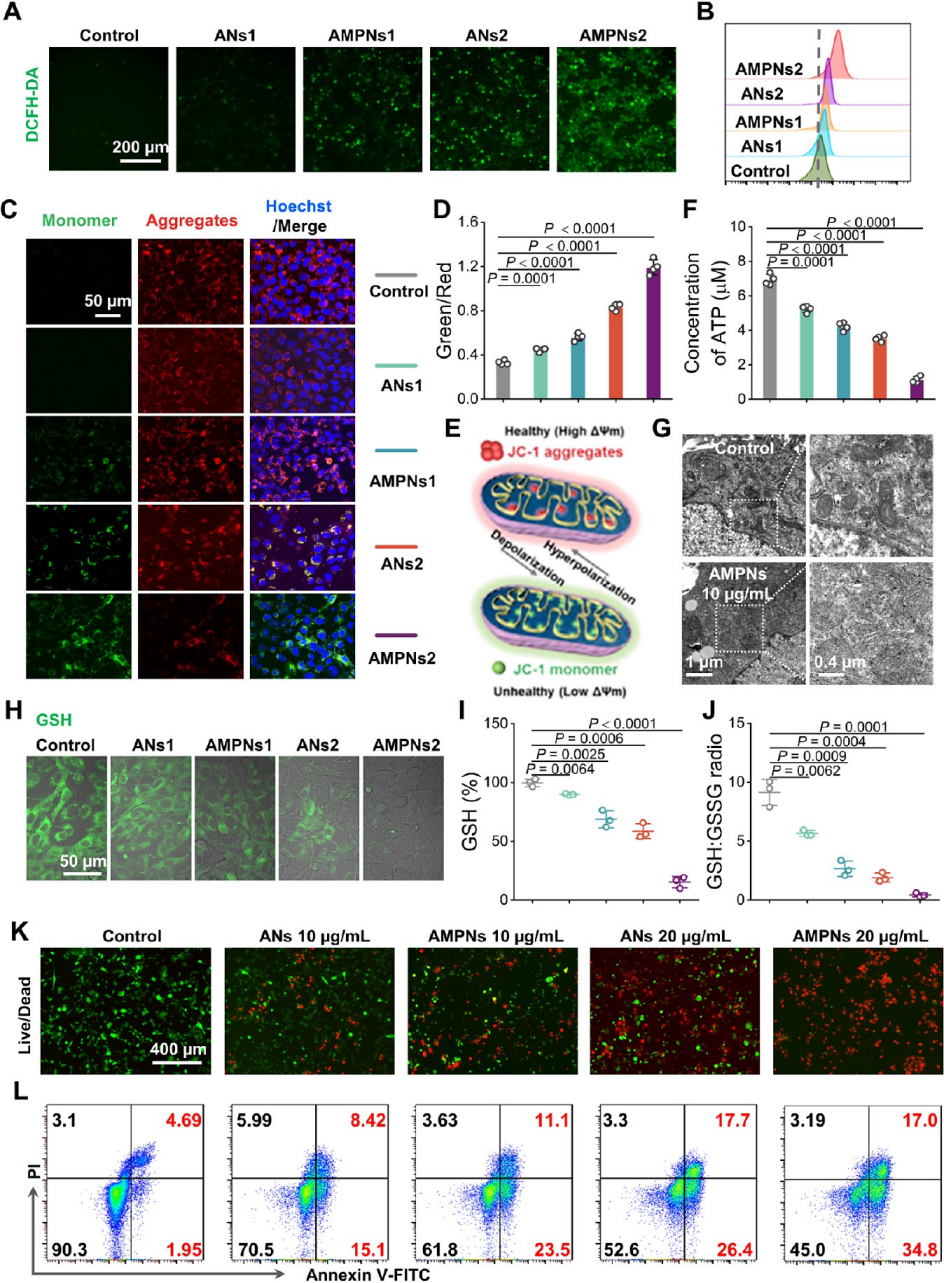
ANs and AMPNs effects on oxidative stress, mitochondrial injury, and apoptosis. (A,B) Cell staining (A) and FC data (B) for ROS production. Scale bars, 200 μm. (C–E) CLSM images (C), quantification (D), and mechanism (E) of JC-1 staining for measurement of mitochondrial depolarization. Scale bars, 50 μm. (F) Intracellular ATP concentrations. (G) Bio-TEM images of mitochondrial damage. (H–J) CLSM images (H) and quantitative analysis of GSH level (I) and the GSH:GSSG ratio (J). Scale bars, 50 μm. (K,L) Live/dead cell staining (K) and FC analysis of apoptosis (L). Scale bars, 400 μm.

We next evaluated the ability of ANs and AMPNs concentrations (5 and 10 μg/mL) to induce ROS production in Hela-6 cells, as measured by fluorescent signal produced by the ROS probe 2′,7′-dichlorofluorescein diacetate (DCFH-DA). This analysis revealed that low and high AMPNs concentrations (AMPNs1 and AMPNs2) consistently have more ROS than the corresponding high and low ANs concentrations (ANs1 and ANs2), with all ANs and AMPNs groups yielding more signal than the PBS control group (Figure 4A), as confirmed by FC analysis of these cells (Figure 4B), likely due to a Fenton reaction initiated by Mn^2+^ released from the degrading AMPNs.

Mitochondria play central roles in energy production and apoptosis and are highly susceptible to the effects of ROS.^45,46^ Hepa1-6 cells incubated with the membrane potential dye JC-1 revealed a robust red fluorescence signal consistent with dye aggregation as a result of high mitochondrial membrane potential (ΔΨm), whereas there was a gradual increase in green fluorescent signals in ANs- and AMPNs-treated cells that was consistent with ROS-induced mitochondrial damage, reduction in ΔΨm, and JC-1 dye disaggregation (Figure 4C–E), which was associated with a decline in ATP and altered mitochondrial morphology (Figure 4F,G). Notably, AMPNs-induced ΔΨm and ATP decreases in HUVEC were negligible, corresponding to the expected low GSH and H_2_O_2_ concentrations in these cells (Figures S17 and S18).

Depleting TME GSH levels is a crucial approach used to enhance the efficacy of ROS-based therapies.^35,47,48^ Hepa1-6 cells incubated with ANs or AMPNs revealed concentration- and time-dependent decreases in green-fluorescent signal, reflecting substantial GSH decreases. The GSH signal was highly depleted after a 12 h AMPNs incubation (Figure S19A), as corroborated by a significant reduction in the GSH percentage and the ratio of GSH to oxidized glutathione disulfide (GSSG) (Figure S19B,C). Surprisingly, both ANs and AMPNs treatment increased GSH consumption (Figure 4H–J), although the ANs-mediated effect was more modest and likely due to As_*x*_O_*y*_ binding to thiolated glutathione peroxidase to block GSSG conversion to GSH,^35,38^ where GSH depletion by AMPNs likely occurred primarily by GSH reaction with MnO_2_ on its surface. AMPNs ROS production and GSH consumption properties suggest that these particles could aid in or mediate ROS-based cytotoxic tumor therapies.

To evaluate the cytotoxic effect of cell counting kit-8 (CCK-8), the assay was first used to evaluate the effects of different concentrations of AMPNs on the viability of Hepa1-6, H22, and HepG2 cells. When the Hepa1-6, H22, and HepG2 cells cultures revealed 20.4 ± 2.9% and 17.5 ± 2.7% cell viability, respectively, after incubation with 15 μg/mL AMPNs (Figure S20) versus cultures spiked with the PBS carrier (90.3%), while Hepa1-6 cultures revealed differential viability decreases when incubated with 10 μg/mL or 15 μg/mL ANs (70.5% and 52.6%) or AMPNs (61.8% and 45%) concentrations (Figure 4K,L), with the greatest difference observed in the size of preapoptotic cell (PI-Annexin V) population observed the ANs- vs AMPNs-treated cells. These findings indicate that AMPNs induced more ROS production, GSH depletion, mitochondrial depolarization, and apoptosis than equivalent ANs doses.

### Upregulation of PD-L1 and Inhibition of Metastasis by AMPNs Degradation Pin1

**Figure 5:**
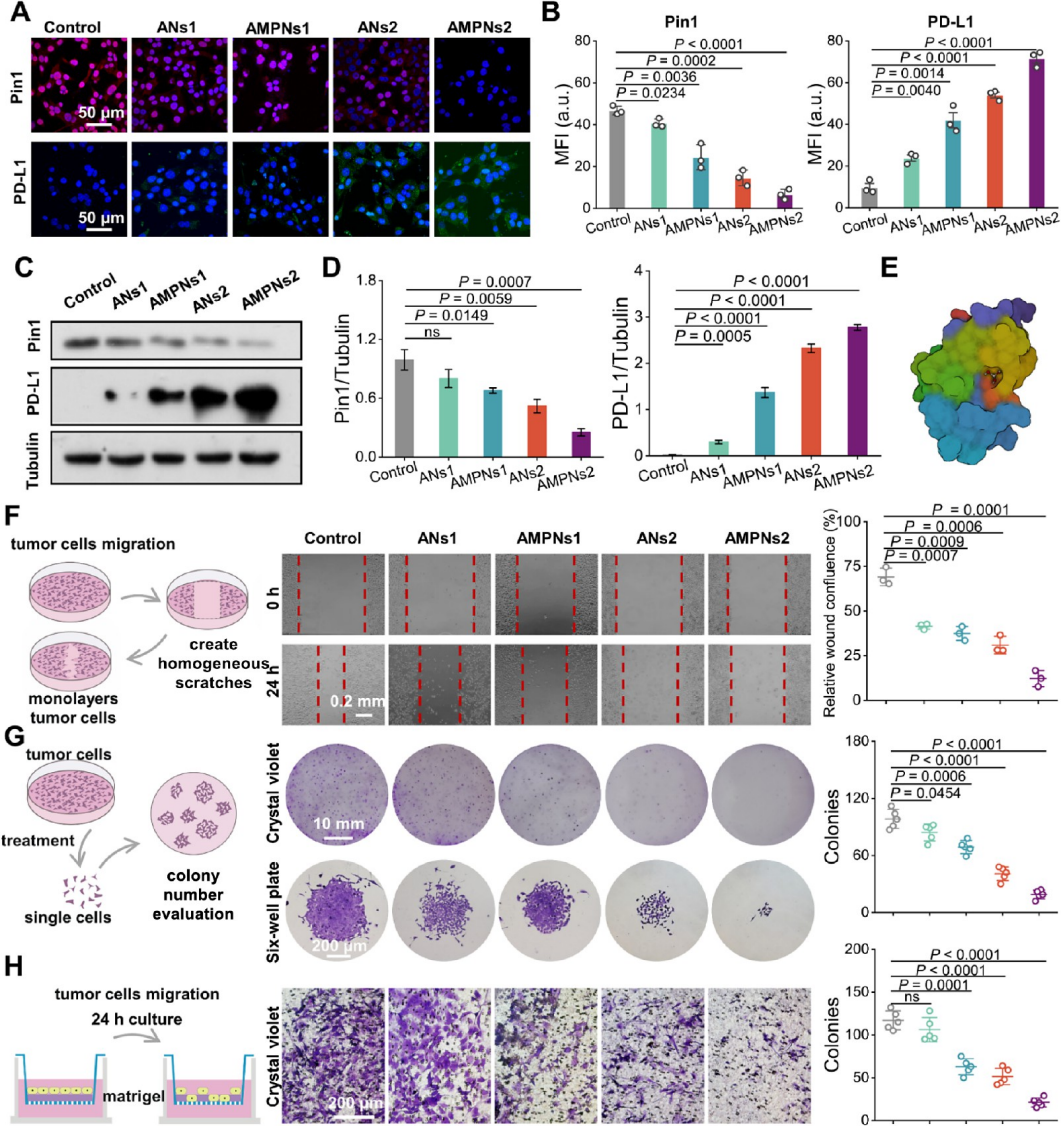
ANs and AMPNs effects on Pin1 and PD-L1 and migration, proliferation, and invasion. (A, B) Immunofluorescence (A) and MFI data (B) for Pin1 (red) and PD-L1 (green). Scale bars, 50 μm. (C, D) Western blot data (C) and graph (D) of Pin1 and PD-L1 expression. (E) Molecular docking simulation As_*x*_O_*y*_ (As_2_O_3_ example) with Pin1. (F–H) Experiment schematics, representative images, and graphs of Hepa1-6 cell migration (F), proliferation (G), and invasion (H) after the indicated treatments.

As_2_O_3_ is reported to attenuate HCC proliferation and invasion via a mechanism that requires Pin1 degradation.^22,25^ We thus hypothesized that AMPNs-derived As_*x*_O_*y*_ could also induce Pin1 degradation in the TME. Consistent with an As-mediated Pin1 degradation effect, Hepa1-6 cells incubated with ANs and AMPNs fractions containing 2.5 and 5.0 μg/mL arsenene revealed reduced Pin1 expression versus that of PBS-treated controls (Figures 5A and S21). The Pin1 mean fluorescence intensity (MFI) of PBS-treated Hepa1-6 cells (46.8 ± 1.7) at 48 h postincubation was 2.2 and 7.1 higher than in the ANs2-treated (21.4 ± 4.9) and AMPNs2-treated (6.6 ± 2.0) cultures (Figure 5B) similar to Pin1 western blot and ELISA results (Figures 5C and S22). These cells also revealed PD-L1 increases corresponding to the observed Pin1 decreases, and these results were mirrored by MFI (9.8 ± 2.6 and 71.6 ± 3.7) and western blot results (0.02 ± 0.01 and 2.8 ± 0.06) (Figure 5A–D). Pin1 degradation results observed in these experiments were consistent with a potential direct effect of As_*x*_O_*y*_ on Pin1 degradation since a molecular docking simulation revealed that As_2_O_3_ could directly interact with a pocket on Pin1 (Figure 5E). Moreover, the direct interaction between As_2_O_3_ and Pin1 did not yield any significant alteration in the mRNA level of Pin1 (Figure S23).

We next evaluated the effect of ANs and AMPNs on As-mediated Pin1 inhibition to attenuate Hepa1-6 migration, proliferation, and invasion.^20,24^ Consistent with reduced Pin1 inhibition, greater cell migration areas were observed in ANs2- versus AMPNs2-treated Hepa1-6 cultures (30.9 ± 4.0 versus 12.2 ± 3.7%; Figure 5F). Similarly, in the colony formation assay used to evaluate cell proliferation, differential decreases were observed in the number of colonies (40.8 ± 6.4 versus 19.0 ± 4.1) that grew on the ANs2-treated versus AMPNs2-treated cells (Figure 5G). AMPNs2-treated cells also revealed less invasive ability than ANs2-treated cells, as determined by the number of cells that migrated through a matrigel matrix after 24 h (51.4 ± 8.6 versus 21.2 ± 4.7) in these cultures (Figure 5H). These findings indicated that in Hepa1-6 cells treated with equal doses of ANs and AMPNs, AMPNs had superior performance to inhibit Pin1 and increase PD-L1 expression, and attenuate cell migration, proliferation, and invasion, suggesting their increased potential utility to enhance ICI therapy for HCC.

### Evaluation of the In Vivo Characteristics of AMPNs

**Figure 6:**
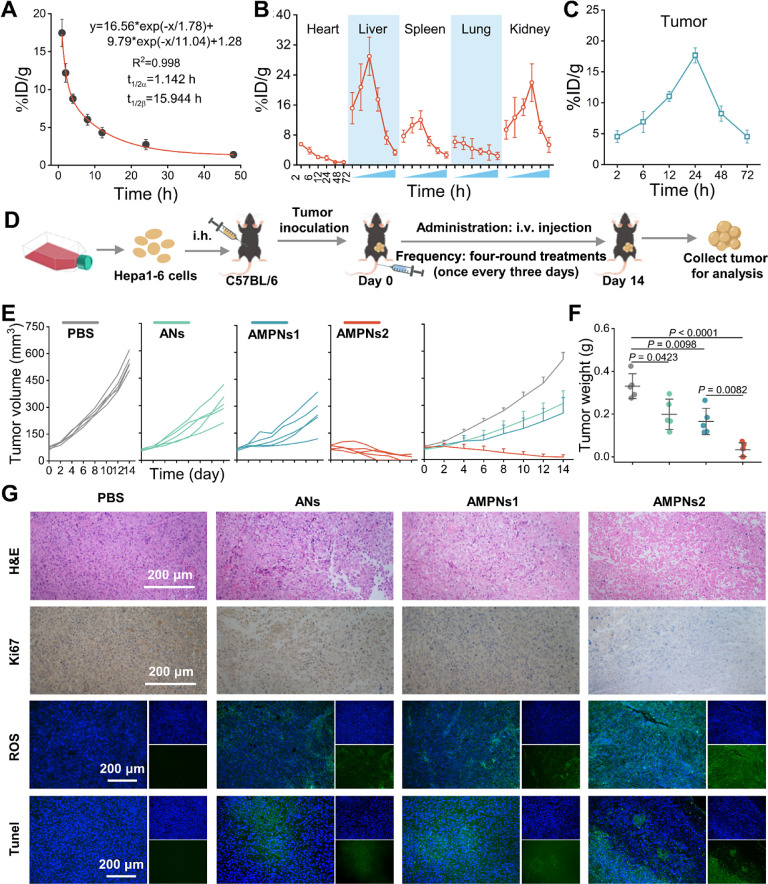
ANs and AMPNs antitumor effects in a mouse model of HCC. (A–C) AMPNs clearance rate from the circulation (A), accumulation in major organs (B), and tumor tissue (C) over time. (D) Schematic of the HCC tumor model and AMPNs treatment procedure. (E) Tumor growth curves. (F) Day 14 excised tumor weights. (G) H&E, Ki67, ROS, and TUNEL histology images for tumor tissue of ANs- and AMPNs-treated mice. Scale bars, 200 μm.

As_2_O_3_ treatment can attenuate tumor growth at the cost of substantial side effects; however, AMPNs characteristics may permit effective treatment without these detrimental effects.^38,43,49^ To evaluate the potential in vivo side effects of systemic AMPNs administration for HCC treatment, we first analyzed the effect that increasing AMPNs doses had on blood samples and a hemolysis rate (2.9 ± 0.6%) that was below the accepted 5.0% limit, suggesting the potential for intravenous AMPNs delivery (Figure S24). To investigate long-term toxicity, mice were intravenously injected with four AMPNs doses (10 mg/kg arsenene every 3 days) and monitored for body weight losses and other standard indicators of drug safety. All of these parameters fell within their acceptable ranges, suggesting that AMPNs treatment did not induce substantial inflammation or injury in the analyzed organs (Figure S25A–F). Next, to evaluate the APMNs pharmacokinetics and biodistribution in mice bearing HCC tumors, these mice were iv-injected with AMPNs and sacrificed at serial time points to analyze AMPNs levels in the blood, tumor, heart, liver, spleen, lung, and kidney tissue by ICP-MS. The blood circulation of the two-chamber model revealed an approximate 1.4 h half-life for AMPNs in the circulation (Figure 6A). AMPNs were enriched in all of the highly perfused tissues analyzed with the greatest enrichment detected in liver tissue (29.0 ± 5.1% injected dose/gram tissue) at 12 h postinjection, with a rapid and progressive enrichment detected in most tissues after this point (Figure 6B). Notably, similar robust APMNs accumulation (17.7 ± 1.2% ID/g) and kinetics were also observed in HCC tumor tissue (Figure 6C).

Next, to investigate in vivo therapeutic potential, mice with ∼75 mm^3^ HCC tumor volumes were randomly assigned to four groups that were intravenously injected with PBS, ANs at a 5 mg/kg arsenene dose, or AMPNs at 2.5 or 5 mg/kg arsenene doses (AMPNs1 and AMPNs2) at days 0, 3, 6, and 9 and sacrificed on day 14 to collect tumors and major organs for analysis (Figure 6D). HCC tumor growth rates rapidly increased in the PBS-injected group but were attenuated to different extents in the ANs- and AMPNs-treated groups, all of which revealed significant decreases in tumor growth (Figure 6E). Notably, ANs- and AMPNs1-injected mice revealed similar tumor attenuation even despite the difference in the arsenene dose between these groups (5 versus 2.5 mg/kg), while most of the AMPNs-treated mice revealed progressive decreases in tumor volume over the treatment time course, with half of these mice demonstrating tumor clearance, to achieve an overall 97.5% inhibition rate. Similar results were also observed when the final tumor weights were observed in these mice (Figures 6F and S26). These differences corresponded with decreased cell proliferation and increased ROS production and apoptosis, as detected in representative HCC tumor sections by Ki67, DCFH-DA, and TUNEL staining (Figures 6G and S27), with similar changes observed in the ANs- and AMPNs1-treated groups and marked changes observed in the AMPNs-treated group. No differences in body weight or organ pathology were observed between the PBS and ANs- or AMPNs-treated groups (Figures S28 and S29), however, indicating that significant therapeutic effects were produced in the absence of detectable systemic toxicity.

### AMPNs Reshape TIME by Inhibiting Pin1

**Figure 7:**
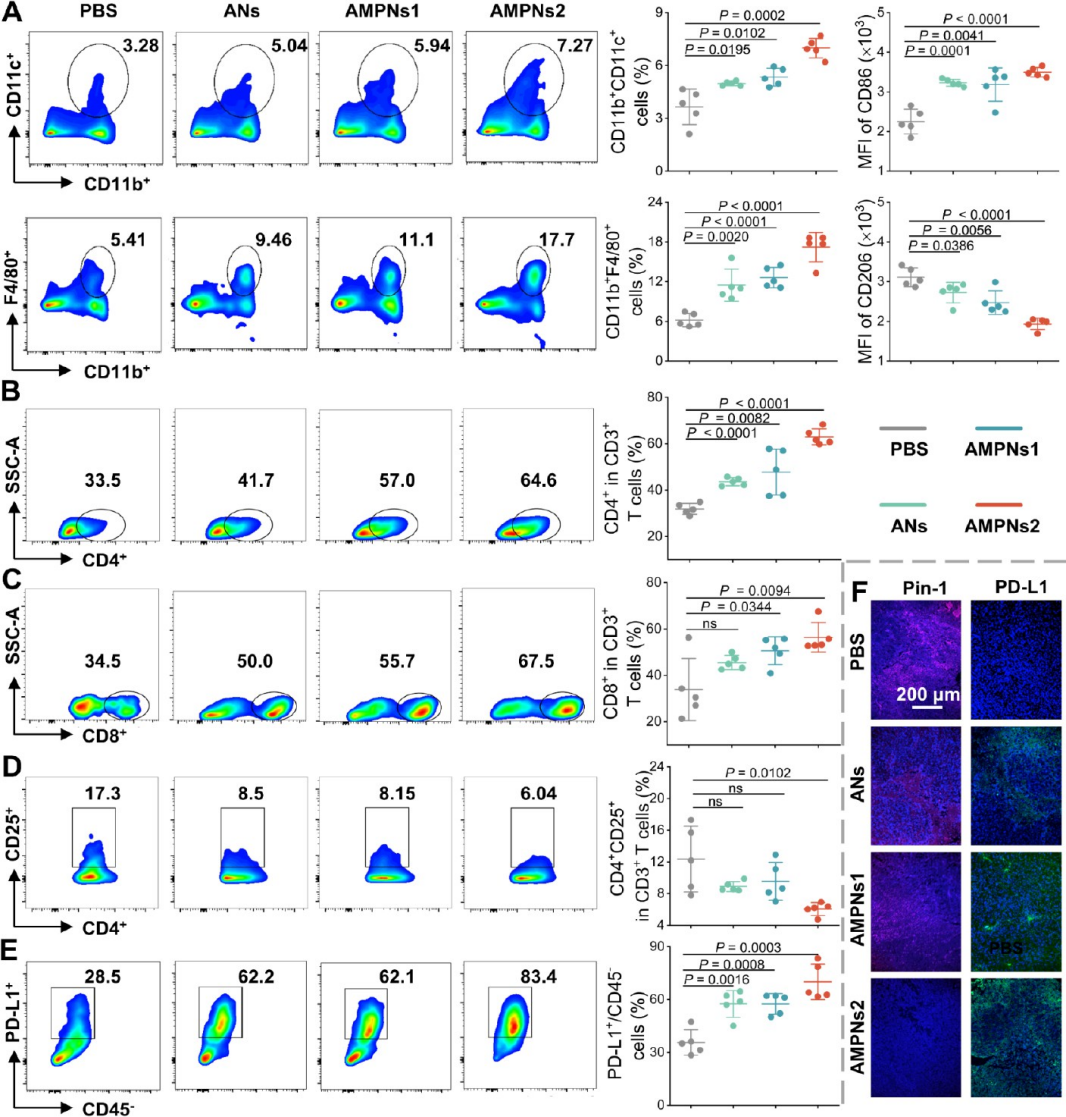
AMPNs effects on the TIME of the HCC mouse model. (A–D) Representative FC analysis data and aggregate graphs for TME DCs, macrophages (A), CD4^+^ T cells (B), CD8^+^ T cells (C), and Treg cells (D) after treatment with or without ANs or AMPNs. (E) Representative FC analysis data and aggregate graphs for PD-L1^+^ tumor cells after treatment with or without ANs or AMPNs. (F) Representative immunohistology of Pin1 and PD-L1 expression in mouse tumor tissue. Scale bars, 200 μm.

To investigate the effects of ANs and AMPNs on the TIME, we used FC analysis to evaluate changes in tumor-infiltrating immune cells (Figure S30). AMPNs2 treatment increased dendritic cells (DCs) and macrophage frequency (7.3% and 17.7%) versus PBS-treated mice (3.3% and 5.4%), with smaller increases (5.0–5.9% and 9.5–11.1%) observed in ANs- and AMPNs1-treated mice (Figure 7A). MHCI expression, which directly regulates antitumor immune responses, was also increased 1.6- and 1.4-fold in the tumor-derived DCs and macrophages of AMPNs2-treated versus the PBS-treated mice (Figure S31A,B), and similar MHCII expression differences were observed in these groups (Figure S31C,D). ANs/AMPNs1 and AMPNs2 treatment was also associated with M1 macrophage increases and M2 macrophage decreases consistent with a decrease in the tumor promotion potential of the TIME (Figure 7A). All ANs and AMPNs-treated mice also revealed TIME increases in both CD4^+^ and CD8^+^ T cells, with the largest increases observed in the AMPNs2-treated group (Figure 7B,C). Notably, these increases were matched by corresponding decreases in the abundance of immunosuppressive Tregs (Figure 7D) and increases in immunostimulatory PD-L1-positive immune cells (Figure 7E), consistent with a tumor-suppressive TIME phenotype, as were increases in granzyme B^+^ CD8^+^ T cells and TNF-α^+^ and IFN-γ^+^ CD4^+^ T cells (Figure S32). Both treatments also decreased tumor Pin1 expression as measured by immunofluorescence and ELISA results (Figures 7F, S33A and S34), and had inverse effects to increase the frequency of PD-L1^+^ cells in the TMEs of these mice (Figures 7F and S33B). Taken together, these results indicate that AMPNs treatment induces responses that remodeled the TIME and TME to promote tumor inhibition or clearance, including via mechanisms that increased PD-L1 expression by increasing Pin1 degradation.

### Combination Therapy Inhibits Tumor Recurrence

AMPNs treatment induced PD-L1 increases that could increase the response rate to anti-PD-L1 or anti-PD-1 therapy by increasing tumor-directed immune responses to enhance the elimination of primary tumors and prevent tumor recurrence.^10,50^

We therefore analyzed the effect of AMPNs treatment on tumor development in mice injected with a high dose of Hepa1-6 cells in the right dorsal region to produce similar size tumors, then treated with PBS, anti-PD-L1 antibodies, AMPNs, or both AMPNs and anti-PD-L1 antibodies, and finally reinjected with a high dose of Hepa1-6 cells in the left dorsal region to mimic tumor recurrence after treatment (Figure S35A). In this study, compared to anti-PD-L1 or AMPNs treatment (37.6 and 82.5% inhibition), AMPNs plus anti-PD-L1 prevented tumor growth or eliminated existing tumors in all mice (Figure S35B). Notably, after rechallenge with a second dose of Hepa1-6 in the left dorsal region, none of the mice previously treated with AMPNs and anti-PD-L1 showed detectable tumor growth (Figure S35C). Similar results were also observed when final tumor weights were observed in these mice (Figures S35D,E) and corresponded with tissue staining and TME Pin1 inhibition (Figure S36). FC analysis of the tumor-infiltrating immune cells of these mice revealed profound increases in DCs, macrophage, and CD4^+^ and CD8^+^ T cell abundance (Figure S37). Combined therapy also significantly increased the abundance of granzyme B^+^ CD8^+^ T cells and TNF-α^+^ CD4^+^ T cells (Figure S38), decreased the abundance of naive T cells (T_N_; CD44^–^ CD62L^+^), and significantly increased the abundance of central memory T cells (T_CM_; CD44^+^ CD62L^+^) (Figure S39).

**Figure 8:**
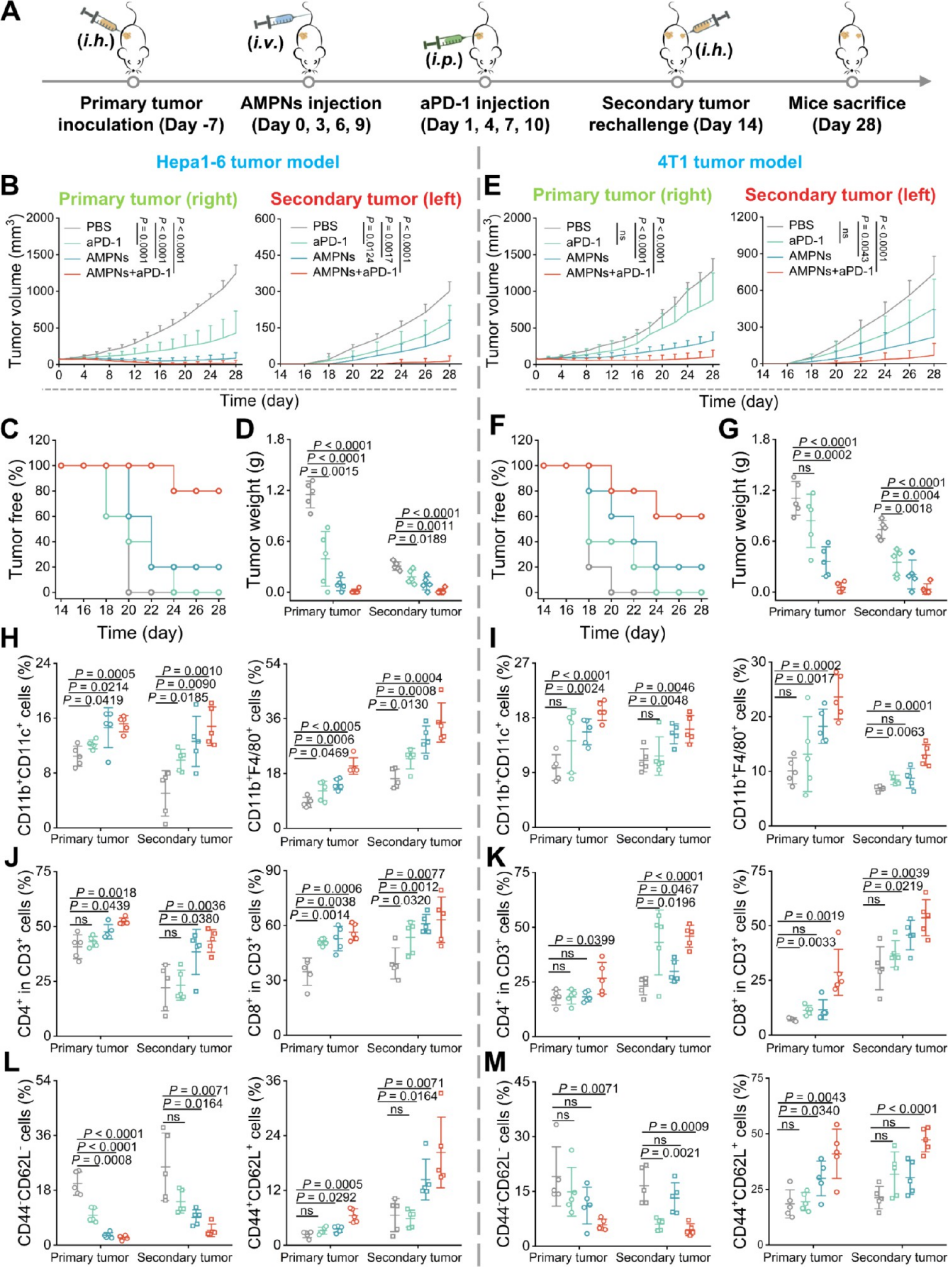
Combination therapy inhibits tumor recurrence. (A) Schematic of the experimental design. (B) Average tumor growth curves of primary and secondary Hepa1-6 tumors. (C) Tumor-free survival curves of the secondary Hepa1-6 tumor. (D) Tumor weight of primary and secondary Hepa1-6 tumors. (E) Average tumor growth curves of primary and secondary 4T1 tumors. (F) Tumor-free survival curves of the secondary 4T1 tumor. (G) Tumor weight of primary and secondary 4T1 tumors. (H,I) DCs and macrophage of abundance in Hepa1-6 tumors (H) and 4T1 tumor (I). (J,K) The quantification results of CD4^+^ and CD8^+^ T cells with the primary and distant tumors. (L,M) FC analysis data for TN and TCM in Hepa1-6 tumors (L) and 4T1 tumors (M).

Besides anti-PD-L1 antibodies, anti-PD-1, which is more widely used in clinical treatment, was selected to evaluate the efficacy of Hepa1-6 tumors (Figure 8A). As expected, the combination of AMPNs and anti-PD-1 demonstrated the successful eradication of four primary tumors without the risk of recurrence, which was more effective than the use of AMPNs alone (Figures 8B and S40A). For the secondary tumor, all the mice previously treated with PBS developed rapid tumor development at this site, and similar results were observed in mice previously treated with anti-PD-1 alone, although individual mice in this group revealed attenuated tumor growth. The majority of the mice previously treated with APMNs did not develop tumor or revealed attenuated tumor growth rates (80% recurrence); however, only one mouse previously treated with AMPNs and anti-PD-1 revealed detectable tumor growth. Combined therapy thus produced a remarkable protective effect that enabled treated mice to effectively resist a second challenge (Figures 8C and S40B). The effectiveness of combined AMPNs and anti-PD-1 treatment was also supported by tumor weight data and tissue staining of these mice at sacrifice and corresponded with TME Pin1 inhibition and TIME changes (Figures 8D and S40C,D).

In addition to the Hepa1-6 tumor model, we further evaluated the efficacy of this combination therapy in the 4T1 tumor model, which exhibits much lower immunogenicity than the Hepa1-6 tumor and high Pin1 expression. We assessed the cytotoxicity of AMPNs against 4T1 cells in vitro. As anticipated, AMPNs effectively elicited oxidative stress, mitochondrial impairment, and ATP depletion in 4T1 cells (Figure S41A-C). Notably, AMPNs also exhibited inhibitory effects on the metastasis and invasion of 4T1 cells (Figure S41D). Next, we discovered that anti-PD-1 had weak suppressive effects on 4T1 tumors, and AMPNs had strong inhibition of primary tumors but no significant inhibition of secondary tumors (Figures 8E and S42A,B). Significantly, the combination treatment showed greater benefit than either AMPNs or anti-PD-1 alone, inhibiting or eradicating the primary tumor and preventing the recurrence of secondary implanted tumors of the mice (40% recurrence) (Figure 8F,G). Tissue staining revealed tumor damage, Pin1 inhibition, and increased PD-L1 expression (Figure S42C,D).

To explore the mechanism of the synergistic antitumor effect induced by AMPNs in combination with anti-PD-1, the TIME of the primary and secondary tumors was analyzed by FC. As expected, the frequency of DCs and macrophages in primary tumors was also increased 1.7- and 2.8-fold in TIME of combined treatment-treated versus the PBS-treated mice, and similar results were observed in secondary tumors (Figures 8H and S43A,B). For the 4T1 tumor model, the combination of AMPNs with anti-PD-1 treatment also increased quantities of DCs and macrophages in the primary and secondary tumors compared to AMPNs alone (Figures 8I and S43C,D). For both primary and secondary tumors in Hepa1-6 and 4T1 tumor models, all mice treated with AMPNs and anti-PD-1 alone demonstrated an increasing fraction of CD4^+^ and CD8^+^ T cells in TIME, with the largest increase in the AMPNs + anti-PD-1 group (Figures 8J,K and S44). It was found that treatment with AMPNs + anti-PD-1 also reduced the fraction of TN and increased the abundance of TCM, indicating that the combined treatment induced a stronger memory response (Figures 8L,M and S45). Combined therapy also significantly increased the abundance of granzyme B^+^ and IFN-γ^+^ CD8^+^ T cells and TNF-α^+^ CD4^+^ T cells (Figure S46), consistent with a tumor-suppressive TIME phenotype. Body weight and H&E staining data for the major organs of mice in all these did not reveal detectable tissue abnormalities, supporting the favorable biosafety profile of all these treatments (Figure S47). These data indicate that AMPN and anti-PD-L1 combination therapy could effectively enhance the response rate of ICI, successfully inhibiting the growth and preventing the recurrence of HCC and breast cancer.

## Discussion

HCC treatment strategies are severely limited by the heterogeneity of HCC and its TIME and low response rates, which are a persistent challenge. Low PD-L1 expression may contribute to the variable response to ICI therapy, and it is thus important to factor this issue into the decision to administer ICI therapy. Pin1 appears likely to play an important role in the ICI response of HCC tumors since it regulates numerous signaling pathways that are critical for tumor growth and progression.^14,17−20^ As_2_O_3_ can promote Pin1 degradation, which could increase ICI efficacy in treatment of HCC tumors, but has substantial toxicity that limits it potential utility in therapeutic approaches.^20,22−25^ The results we present in this study, however, indicate that AMPNs can effectively inhibit HCC tumor Pin1 expression to increase TIME PD-L1 expression while eliminating systemic toxicity associated with As_2_O_3_ treatment at doses that promote HCC tumor regression and prevent tumor recurrence after a subsequent high-dose HCC challenge.

As_2_O_3_ treatment has promising results to treat mouse models of HCC but has substantial side effects.^22,33,37,46^ AMPNs used in this study did not exhibit significant in vivo toxicity profiles at therapeutically effective doses in our mouse HCC model experiments, however, suggesting that a broad AMPNs dose range could be used to safely treat HCC. Arsenene-based nanomaterials have been evaluated as potential cancer therapeutics in other studies,^38,51^ but these differed from the current study, which did not employ an “AND gate” approach to limit arsenene conversion outside the TME but instead used surface modifications to enhance their stability, biocompatibility, or tumor-targeting activity, or to delivery cancer therapeutic agents. These studies relied on the ability of their arsenene nanomaterials to selectively induce ROS production in cancer cells to induce mitochondrial and DNA damage that could induce cell-cycle arrest and apoptosis, but in one case, they also employed arsenene nanomaterial to act as photothermal therapy targets to augment their ROS effects.^36,38^ Notably, these studies were performed in nude mice and thus would not account for TIME effects on tumor growth and regression. By contrast, results of this study indicate that AMPNs treatment can modulate the TIME and induce an immune response that can restrict the development of subsequent tumors when previously treated mice are challenged with high Hepa1-6 cell doses that would otherwise cause rapid tumor development.

Multiple factors, including reduced TME PD-L1 expression and TIME changes, can diminish the efficacy of anti-PD-L1/anti-PD-1 therapy.^10,20,52−54^ Interventions to upregulate TME PD-L1 expression or alter the TIME thus hold promise as a means to improve anti-PD-L1/anti-PD-1 therapy response rates.^3,8,10,55^ However, these mechanisms involved can be complex. For example, high TME lactic acid levels have been reported to upregulate PD-L1 expression and suppress antitumor immune responses to promote tumor growth,^52,56^ but nanoparticle-mediated depletion of TME lactic acid was found to improve the response to anti-PD-L1/anti-PD-1 immunotherapy, potentially via effects to reverse lactate-mediated effects to induce M2 macrophage polarization and suppress T and NK cell activation and pro-inflammatory cytokine expression.^53^ Conversely, enhanced tumor glycolysis can increase PD-L1 expression, but nanoparticle-mediated depletion of TME glucose levels via glucose oxidase delivery can also promote PD-L1 expression to enhance the effectiveness of anti-PD-L1 therapy.^10^

In the current study, we used AMPNs that decrease Pin1 expression to upregulate PD-L1 expression since Pin1 is overexpressed throughout HCC development and plays an important role in regulating PD-L1 degradation.^17,20^ Targeted delivery of another Pin1 inhibitor, AG17724, was found to attenuate tumor growth and extend survival in a mouse model of pancreatic ductal carcinoma, although this study did not analyze PD-L1 expression or evaluate the efficacy of anti-PD-L1 therapy.^20^ In this study, however, we found that AMPNs could significantly upregulate PD-L1 expression in the TME and induce a tumor-suppressive TIME profile, consistent with an observed enhanced attenuation of tumor growth and the prevention of tumor recurrence in mice treated with both AMPNs and anti-PD-L1/anti-PD-1 antibodies. However, it should be noted that Pin1 degradation can have multifaceted effects, and further characterization studies, including proteomic analyses, should be performed to evaluate other mechanisms that may regulate PD-L1 expression or other potential pathways that could contribute to the antitumor effects of AMPNs treatment.

Notably, Pin1 overexpression is not limited to HCC and is commonly detected in several other cancers and cancer-associated cell types, including breast cancer and pancreatic ductal carcinoma cells and cancer-associated fibroblasts.^20,22,57^ Our study demonstrated that AMPNs are also effective at increasing the response rate to anti-PD-1 therapy in a mouse model of breast cancer. Similar combination therapy approaches could thus prove useful in these and potentially other solid tumor types that many normally display refractive phenotypes when treated with anti-PD-L1/anti-PD-1 therapy, particularly for those with low intrinsic immunogenicity.

## Conclusions

Through comprehensive bioinformatics analysis and clinical sample studies, we have identified significant upregulation of Pin1 expression in HCC, underscoring its pivotal role in driving cancer progression. Furthermore, targeted inhibition of Pin1 effectively suppresses tumor proliferation and exerts regulatory control over the expression of PD-L1, thereby highlighting the promising potential of Pin1 inhibition as a novel strategy for synergistic immunotherapy. The adverse effects of As_2_O_3_ as a Pin1 inhibitor can be mitigated by fabricating AMPNs controlled by the “AND” gate, which are obtained through liquid-phase exfoliation and surface reduction. AMPNs selectively release As_*x*_O_*y*_ in response to high concentrations of GSH and H_2_O_2_ within the TME, causing oxidative stress and mitochondrial damage. Furthermore, AMPNs degrade Pin1 expression while promoting PD-L1 upregulation in tumor cells, thereby triggering a robust antitumor immune response and enhancing the efficacy of PD-L1/PD-1 immune checkpoint blockade therapy. The intravenous administration of AMPNs in vivo experiments not only effectively suppressed Pin1 expression and inhibited Hepa1-6 tumor growth by 97.5% but also successfully reversed the TIME. Furthermore, combination therapy of AMPNs and anti-PD-1 achieved complete remission in three out of five mice bearing Hepa1-6 tumors. Importantly, even after re-inoculation of Hepa1-6 cells, no tumor recurrence occurred in the combined treatment group, indicating the robust induction of durable immune memory by the combined therapy. In addition to Hepa1-6 tumors, the combination therapy also demonstrated promising therapeutic effects on the 4T1 tumor model with high Pin1 expression. Therefore, the combined treatment of AMPNs and anti-PD-1 antibodies represents a prospective therapeutic strategy for tumors that overexpress Pin1.

## Supporting Information Available

Experimental details, Pin1 and PD-L1 expression, immunofluorescence images, flow cytometry analysis, H&E staining, TEM images, SEM images, AFM images, Raman spectra, FT-IR spectra, XRD, UV–vis absorption spectra, particle size, cumulative release of As ions, cell viabilities, cytotoxicity assessments, intracellular uptake, lysosomal escape, JC-1 staining, quantitative analysis of GSH, mRNA level of Pin1, hemolysis test, safety assessments, tumor images, body weight of mice, and abundance of immune cells (PDF)
