# Supplementary material for: Valence-Change MnO2-Coated Arsenene Nanosheets as a Pin1 Inhibitor for Hepatocellular Carcinoma Treatment
Source: J Am Chem Soc. 2024 Jul 25;146(31):21568–82. doi: 10.1021/jacs.4c05162 (PMC11311233; doi:10.1021/jacs.4c05162)
Supplement: Supplementary file 1 — ja4c05162_si_001.pdf [file ja4c05162_si_001.pdf]

Supporting Information

Valence-Change MnO_2-Coated Arsenene Nanosheets as a Pin1 Inhibitor for Hepatocellular Carcinoma Treatment

Jingguo Wang[1,#], Siping Liang[1,#], Dongdong Zhu[1], Xiaocao Ma[1], Qin Peng[1], Guanzhao Wang[2], Yuting Wang[1], Tiantian Chen[1], Minhao Wu[1,*], Tony Y. Hu[3,*], and Yuanqing Zhang[1,2,*]

[1]Zhongshan School of Medicine, Sun Yat-Sen University, Guangzhou 510080, China
[2]School of Pharmaceutical Sciences, Sun Yat-Sen University, Guangzhou 510006, China
[3]Center of Cellular and Molecular Diagnosis, Tulane University School of Medicine, New Orleans,
[#]These authors contributed equally

*Correspondence: wuminhao@mail.sysu.edu.cn (M.W.); Tonyhu@tulane.edu (T.H.); zhangyq65@mail.sysu.edu.cn (Y.Z.)

Experimental Section

Materials

Arsenene powder and As_2O_3 were purchased from Sigma–Aldrich. Potassium permanganate ($KMnO_4$) was obtained from Aladdin Industrial Co. Ltd. m-PEG- NH_2 and Cy5-PEG-NH_2 were purchased from Xi'an ruixi Biological Technology Co., Ltd. Hoechst-33342, CCK-8 Assay Kit, Calcein-AM/PI Assay Kit, DCFH-DA, and the Mitochondrial membrane potential assay kit, ATP assay kit, GSH and GSSG Assay Kit, and Apoptosis Detection Kit were purchased from Beyotime Biotechnology. ThiolTrace Violet was obtained from AAT Bioquest. Anti-mouse PD-L1 and anti-mouse PD-1 were bought from Bioxcell Company. Antibodies for FC analyses were obtained from BioLegend. HUVEC, HEK293, HepG2 and Hepa1-6 cells were obtained from the Cell Bank of the Chinese Academy of Sciences.

ANs and AMPNs characterization

TEM images and EDS mapping data were obtained using a FEI Tecnai G2 F30 instrument (300 kV). SEM images was captured on a ZEISS Ultra 55 system. AFM images were detected on a Bruker Dimension Fastscan system and

thickness analyses were performed using NanoScope Analysis 1.7 software. Chemical compositions were obtained using XRD (Bruker D8 Advance Davinci) and XPS (Thermo ESCALAB 250) instruments. As ion abundance was measured using an ICP-MS system (Agilent7700). UV-Vis spectra were determined using a UV-2066 UV-Vis spectrophotometer and particle size and ζ potential measurements were measured with a Malvern Zetasizer Ultra instrument. Raman spectra were obtained using an inVia Qontor Raman spectrometer.

ANs and AMPNs synthesis

ANs were prepared using a typical liquid-phase exfoliation method where 50 mg bulk arsenene and 100 mg PEG-NH_2 were dispersed in 50 mL deionized water and sequentially subjected to liquid nitrogen pre-cooling, water bath sonication, and ice-water bath sonication (600 w, 50% power) steps (6 times each for 30 min). ANs were obtained by centrifuging the final mixture at 500 relative centrifugal force (RCF) and 15000 RCF for 10 min, sequentially. ANs were MnO_2-coated by adding 5 mL of a 0.5 mg mL^{-1} $KMnO_4$ solution to 10 ml of 1.0 mg mL^{-1} ANs and mixing this suspension for 60 min at pH 11. MnO_2-coated ANs were pelleted by centrifugation and then mixed with 10 ml of a 2 mg mL^{-1} NH_2-PEG solution overnight, after which AMPNs were recovered by centrifugation and excess NH_2-PEG were removed by sequential deionized water wash steps and AMPNs were stored at 4 °C for subsequent uses.

In vitro arsenic ions release performance

The AMPNs solution (containing 500 μg As) was enclosed in a dialysis bag with a molecular weight cut-off of 7 kDa and immersed in 27 mL of PBS, either with or without GSH, for 6 h. Subsequently, it was transferred to PBS supplemented with H_2O_2. At predetermined intervals, 3 mL of the release buffer was withdrawn and replaced with an equal volume of fresh PBS. The concentration of released arsenic ions was determined using ICP-MS.

AMPNs in vitro cell uptake

AMPNs and ANs cell uptake efficiencies were analyzed by confocal laser scanning microscopy (CLSM) and FC. Hepa1-6 and AML12 cells (1×10^5 cells per well) were seeded in 35 mm glass-bottom culture plates, incubated overnight in a tissue culture incubator, then incubated with 5 μg mL^{-1} Cy5-labeled AMPNs for 0, 1, 2, 4 and 8 h, imaged by CLSM or FC, and these images were analyzed to determine ANs and AMPNs uptake efficiency.

AMPNs in vitro lysosomal escape

Hepa1-6 cells (1×10^5 cells per well) seeded in 35 mm glass-bottom plates for 12 h, incubated with 5 μg mL^{-1} AMPNs for 2, 4 and 8 h, then PBS-washed and incubated with Lyso-Tracker Green for 20 min, and imaged by CLSM.

AMPNs in vitro biocompatibility and cytotoxicity

HUVEC, HEK293 and AML12 cells were seeded in 96-well plates (1×10^4 cells per well) for AMPNs biocompatibility analyses, incubated overnight, and then incubated with 0, 5, 10, 15, 20 and 30 μg mL^{-1} AMPNs concentrations and CCK-8 solution for 1 h before to analyzing dye conversion by a microplate reader to evaluated ANs- and AMPNs-induced changes in cell proliferation. Equivalent Hepa1-6 cell cultures were incubated with increasing ANs and AMPNs concentration and Calcein-AM and propidium iodide to live-dead cell counts as a measure of cytotoxicity.

ROS production and GSH consumption analyses

To evaluate ROS levels, Hepa1-6 cells were treated with 5 or 10 μg mL^{-1} ANs or AMPNs, or PBS carrier, and co-incubated with DCFH-DA for 20 min, PBS-washed, and analyzed by fluorescence microscopy or dispersed by trypsin digestion and collected for FC analysis. To evaluate intracellular GSH levels, Hepa1-6 cells were co-incubated with ANs or AMPNs for 12 h and stained by ThiolTrace Violet for 1 h, PBS washed, and imaged by CLSM. For the quantitative analysis of GSH, Hepa1-6 cells were collected after different treatments and subjected to repeated freeze-thaw cycles to obtain GSH and GSSG. Subsequently, the total glutathione and GSSG contents were determined using a standardized GSH and GSSG Assay Kit (Beyotime Biotechnology) procedure, and the concentration of GSH was calculated using the following formula: GSH = total glutathione – GSSG × 2.

ΔΨm measurements

Hepa1-6 cells (1×10^4 cells per well) seeded in 35 mm glass-bottom plates for 12 h, incubated with 5 or 10 μg mL^{-1} ANs or AMPNs for 12 h, then PBS-washed and incubated with JC-1 for 20 min, and imaged by CLSM or analyzed using a fluorescence microplate reader to quantify the ΔΨm signal.

Immunofluorescence analyses

Hepa1-6 cells (1×10^4 cells per well) seeded in 35 mm glass-bottom dishes were cultured overnight, incubated for 24 h with 5 or 10 μg mL^{-1} ANs or AMPNs, PBS-washed and fixed with 4 % paraformaldehyde. Cells were then treated with 0.2% TritonX-100 and incubated for 0.75 h with 5 % BSA, 12 h with primary antibody (1:500 dilution), and then for 1 h with fluorescence-labeled second antibody (1:1000 dilution), then PBS-washed and imaged by CLSM.

Western blot assay

Hepa1-6 cells subjected to different treatments were lysed in 1× RIPA lysis buffer, the concentration of the resulting protein lysate were analyzed by coomassie brilliant blue, and size fractionated by sodium dodecyl sulfate-polyacrylamide gel electrophoresis, transferred to polyvinylidene difluoride membranes. These membranes were then

blocked for 1 h with BSA (5%), incubated overnight at 4 °C with primary antibody (1:1000 dilution), 1 h with horse radish peroxidase-labeled second antibody (1:2000 dilution), and target protein signal detected using a New-SUPER ECL (KeyGEN) kit was captured.

Wound-healing assay and colony formation

In wound-healing assays, Hepa1-6 cells (1×10^4 cells per well) seeded in six-well plates were cultured overnight, after which sterile 20 μL pipette tips were used to create uniform scratches in the resulting confluent cell monolayers, which were then PBS-washed and incubated with or without 2.5 or 5.0 μg mL^{-1} ANs or AMPNs for 24 h, and imaged by light microscopy before and after this incubation to measure cell migration at the edges of the disrupted monolayer. For colony formation assays, Hepa1-6 cell cultures (1×10^4 cells per well) incubated for 12 h with or without 2.5 or 5 μg mL^{-1} ANs or AMPNs were dissociated by trypsin digestion and seeded into six-well plates (1500 cells per well) and cultured for 10 days. These cultures were then treated with 4 % paraformaldehyde, stained with 0.1 % crystal violet, PBS-washed and analyzed to quantify their colony formation rates.

Cell invasion assay

Hepa1-6 cells were cultured for 12 h to induced serum starvations receiving starvation treatment, dissociated by trypsin digestion, and 1×10^4 cells per well were seeded in the upper chambers of 24-well transwell culture plates containing membranes precoated with Matrigel. Listed ANs or AMPNs concentrations were added to the upper chambers of these plates and DMEM containing 20 % FBS was added to the lower chambers, and cells were cultured for 24 h, after which non-invasive cells were gently wiped away, and invasive cells were fix using 4% paraformaldehyde and stained with 0.1% crystal violet, and five randomly selected areas on membrane were imaged and counted by microscopy.

Animals

C57BL/6 mice (female, 6-8 weeks old) and Babl/c (female, 6-8 weeks old) were purchased from Animal Supply Center of Sun Yat-sen University. All animal experiments were approved by the Ethics Committee Board for Human and Animal Experiments in Zhongshan School of Medicine of Sun Yat-sen University (SYSU-IACUC-2023-B0109). Mice were allowing to acclimatize to the vivarium environment for 1 week prior to the start of all experiments.

AMPNs in vivo safety evaluation

C57BL/6 mice were randomly divided into two groups and injected intravenously with PBS or AMPNs (10 mg/kg) on days 0, 3, 6, and 9, respectively. Mice were sacrificed at day 32 after treatment initiation and major organs and

blood samples were collected for H&E histology, and blood biochemistry and blood routine tests.

AMPNs in vivo pharmacokinetics and biodistribution

AMPNs (2 mg/kg) were injected intravenously into C57BL/6 mice bearing Hela 1-6 tumors ~75 mm^3. Blood samples were collected at 1, 2, 4, 8, 12, 24 and 48 h post-injection and mice were sacrificed to collected major organs and tumors at 2, 6, 12, 24, 48 and 72 h post-injection to evaluate As ions tissue concentrations by ICP-MS.

As_2O_3, ANs, and AMPNs in vivo anti-tumor effects

To establish Hepa1-6 tumor-bearing mice, 1×10^6 Hepa1-6 cells were subcutaneously injected into the right dorsal region of 6-8 weeks old female C57BL/6 mice and resulting tumor volumes ($1/2\times\text{length}\times\text{width}^2$) and body weights were measured every day or every two days. In the As_2O_3 study, mice with ~ 75 mm^3 tumor volumes were randomly divided into two groups that received PBS or As_2O_3 (5 mg/kg), and sacrificed to harvest tumor tissue for FC analysis and Pin1 meaurement after two rounds of injection. In the ANs and AMPNs study, mice were randomly divided into four once their tumor volumes reached ~50 mm^3 and intravenously injected with PBS, or doses of PBS suspensions that contained 5 mg kg^{-1} ANs, 2.5 mg kg^{-1} AMPNs (AMPNs1) or 5 mg kg^{-1} AMPNs (AMPNs2) at days 0, 3, 6 and 9. Mice were sacrificed at day 14 to collect tumors and major organs for subsequent studies, and tumor tissue was analyzed by H&E, immunofluorescence, ROS and TUNEL staining. For the tumor rechallenge experiment, Hepa1-6 tumor-bearing mice and 4T1 tumor-bearing mice with ~75 mm^3 tumor volumes were randomized into four groups that received PBS, therapeutic antibodies (anti-PD-L1 or anti-PD-1; 2.5 mg kg^{-1}), AMPNs (5 mg kg^{-1}), or combined AMPNs + therapeutic antibodies (anti-PD-L1 or anti-PD-1; 2.5 mg kg^{-1}) doses using the same schedule as above, and then rechallenged at day 14 with 4×10^6 Hepa1-6 cells or 4T1 cells injected into the left dorsal flank on day 14, evaluated every 2 days to assess tumor volume, and sacrificed at day 28 days to collect tumors for subsequent experiments.

Flow cytometry analyses

Dissected tumors were diced, digested incollagenase for 1 h, centrifuged, and then suspended in PBS to obtain single-cell suspensions that were incubated for 45 min with fluorescence-conjugated antibodies to CD3 (100218), CD45 (103108), CD4 (100406), CD8 (100714), CD11b (101206), CD11c (117318), F4/80 (123116), CD80 (104722), CD86 (105014), CD25 (102016), CD44 (103032), CD62L (104428), PD-L1 (124308) purchased from BioLegend, then PBS-washed, and analyzed by flow cytometry (BD FACSVerse, BD Biosciences).

For intracellular cytokine analyses, cells suspended in DMEM supplemented with 10% FBS were simulated for 4

h with a PMA (100 nM)/Ionomycin (1 μg/mL)/Brefeldin A (1 μg/mL) cocktail, PBS-washed, stained with surface antibodies for 45 min, incubated with fixation buffer, and then incubated for 45 min with fluorescent antibodies to detect intracellular IFN-γ (505808), TNF-α (506306) and granzyme B (515408), then PBS-washed, fixed by an 45 min incubation with 1% paraformaldehyde, PBS-washed twice, and analyzed by flow cytometry (BD FACSVerse, BD Biosciences). All FC data analysis was performed using FlowJo software (Tree Star Software, San Carlos, California, USA).

Bioinformatics analysis

The RNAseq data (level 3) and corresponding clinical information of 419 HCC were acquired from TCGA dataset (https://portal.gdc.com).

Clinical sample collection and ethic statements

Tumor specimens from surgical resections were obtained from 4 HCC patients in the First Affiliated Hospital of Sun Yat-sen University from 2020 to 2022. The patients' clinical information was shown in Supplementary Table S1. The study was approved with the patient's informed consent and ethical approval (FAH-SYSU2018[43]and ZSSOM-2021[077]).

The tissues were stained with fluorescent dyes after removing the wax and rehydrating the paraffin sections. To retrieve antigens, a citrate-based solution were autoclaved for 10 minutes. Following rehydration, samples were exposed to Triton X-100 at room temperature to enhance permeability. Subsequently, it was blocked with BSA and incubated overnight at 4°C using Pin1 antibody and PD-L1 antibody. The tissues were incubated with secondary antibodies at room temperature for 1 hour, followed by three washes in PBS containing 0.1% Tween 20 for 5 minutes each, and finally mounted using DAPI-containing mounting media.

ID	Age	Gender	Pathology
1	62	male	Hepatocellular carcinoma
2	42	male	Hepatocellular carcinoma
3	75	male	Hepatocellular carcinoma
4	58	female	Hepatocellular carcinoma
5	71	male	Hepatocellular carcinoma
6	31	female	Hepatocellular carcinoma
7	39	male	Hepatocellular carcinoma
8	55	male	Hepatocellular carcinoma
9	46	male	Hepatocellular carcinoma
10	61	female	Hepatocellular carcinoma
11	40	male	Hepatocellular carcinoma
12	78	male	Hepatocellular carcinoma

Table S1. Summary of Hepatocellular carcinoma patients.

Statistical analysis

All data are presented as mean ± SD values. Statistical significance of potential differences between groups were analyzed using two-tailed Student's t-tests in using Microsoft Excel 2016 software. A P value < 0.05 represented a statistically significant difference.

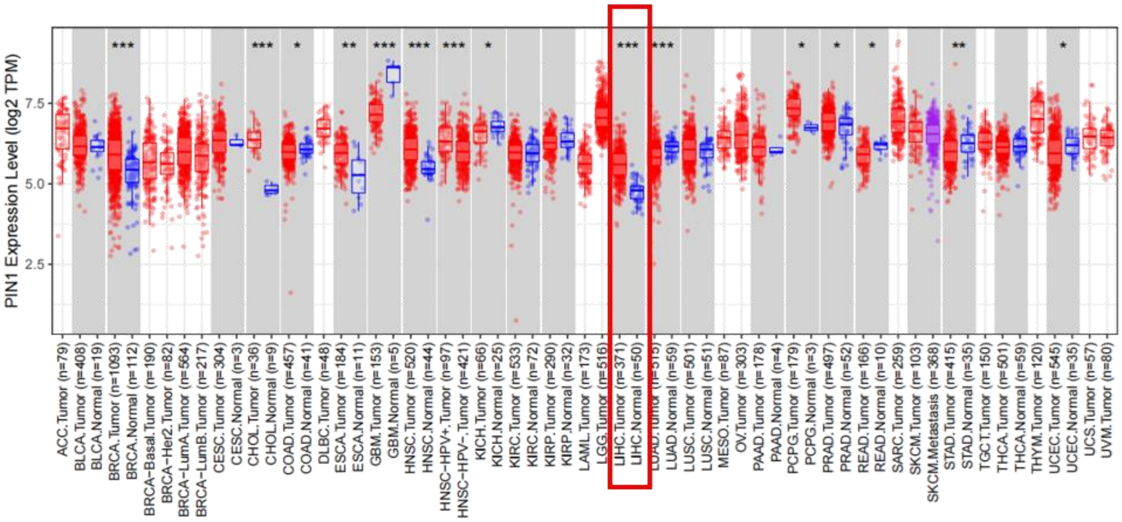

Figure S1. Pin1 expression in tumor and normal tissues.

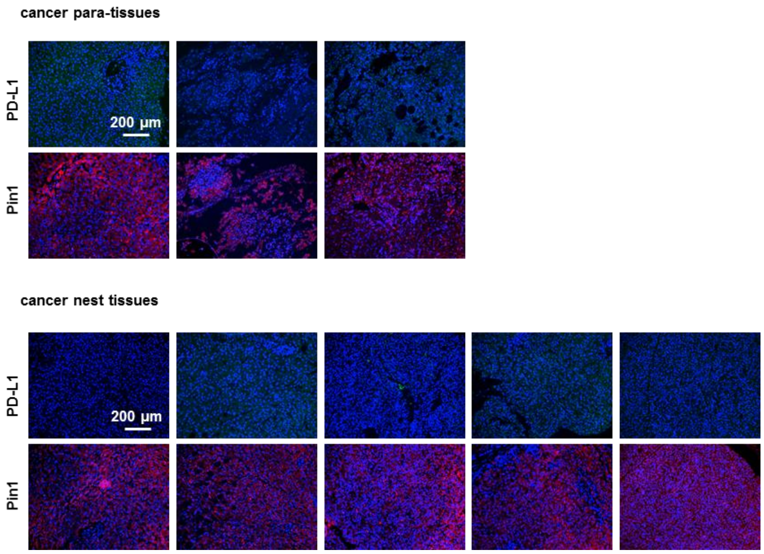

Figure S2. Immunofluorescence staining of HCC para-tumor and cancer nest tissues. (PD-L1: green; Pin1: red). Scale bars, 200 μm.

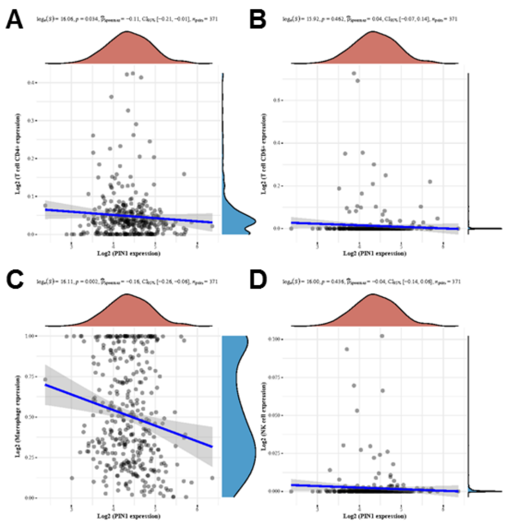

Figure S3. Spearman correlations between Pin1 expression with (A) $CD4^+$ T cells, (B) $CD8^+$ T cells, (C) macrophage and (D) NK cells.

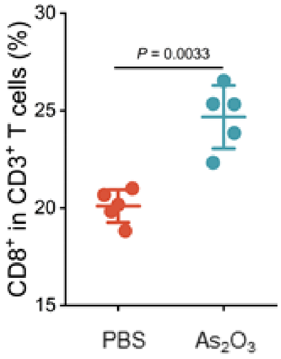

Figure S4. $CD8^+$ T cells frequencies in tumors.

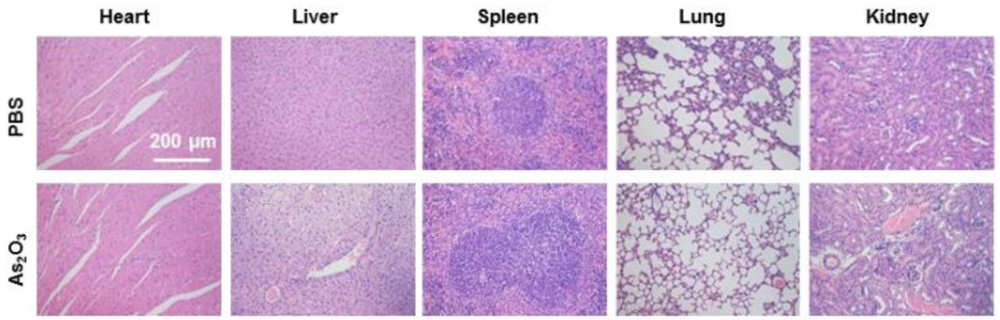

Figure S5. H&E staining images after treatment with PBS or As_2O_3.

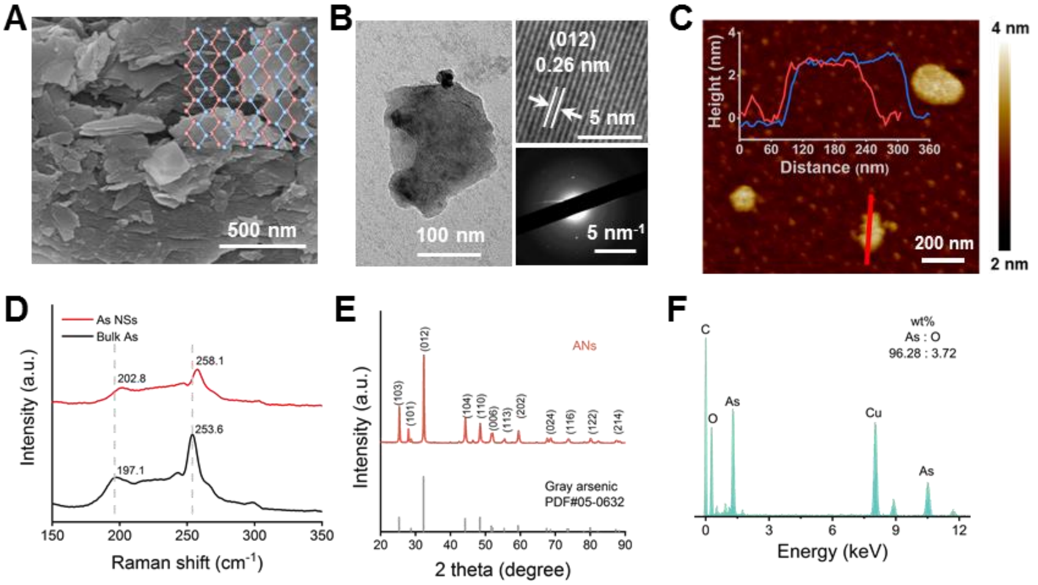

Figure S6. (A) The SEM image and geometrical structures of arsenene powder. (B) TEM, HRTEM and SAED image of ANs. (C) AFM images and the corresponding thickness profiles of ANs. (D) Raman spectra, (E) XRD and (F) EDS analysis of ANs.

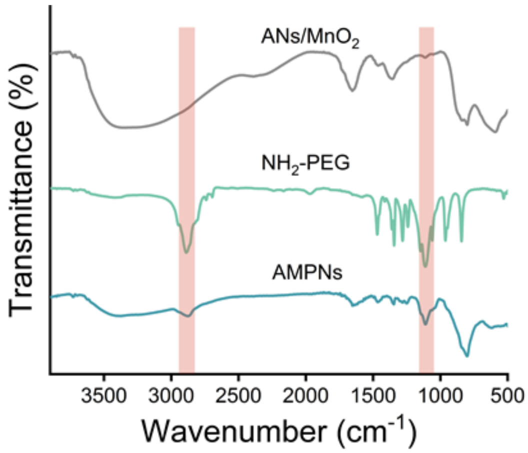

Figure S7. FTIR spectra of ANs/MnO_2, NH_2-PEG, and AMPNs.

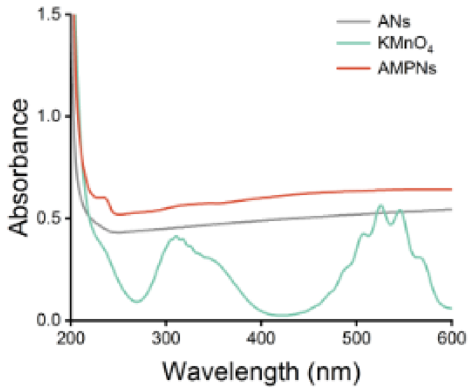

Figure S8. UV–vis spectra of ANs, $KMnO_4$, and AMPNs.

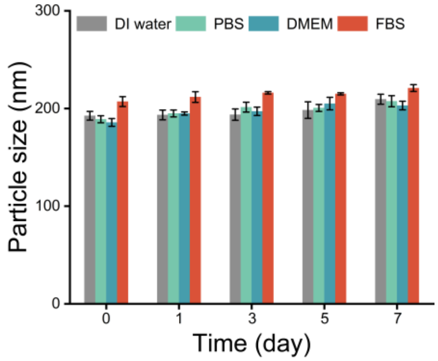

Figure S9. Particle size of AMPNs after incubation with different solvents for varying time intervals.

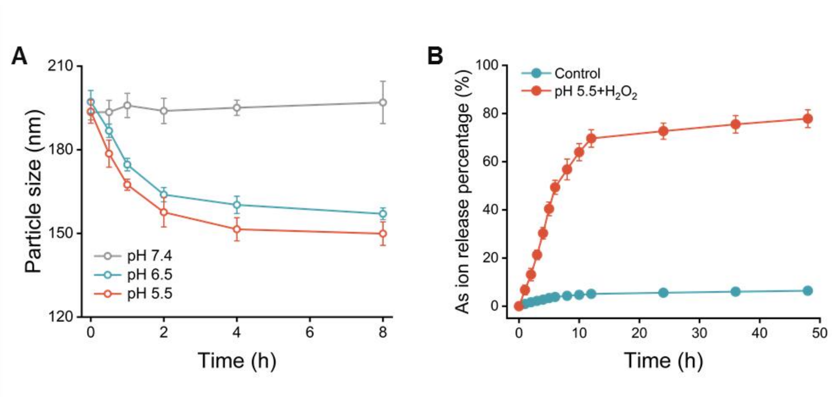

Figure S10. (A) Particle size of AMPNs after incubation with different pH for varying time intervals. (B) Cumulative release profiles of As ions from AMPNs with H_2O_2 and PBS (pH 5.5) exposure.

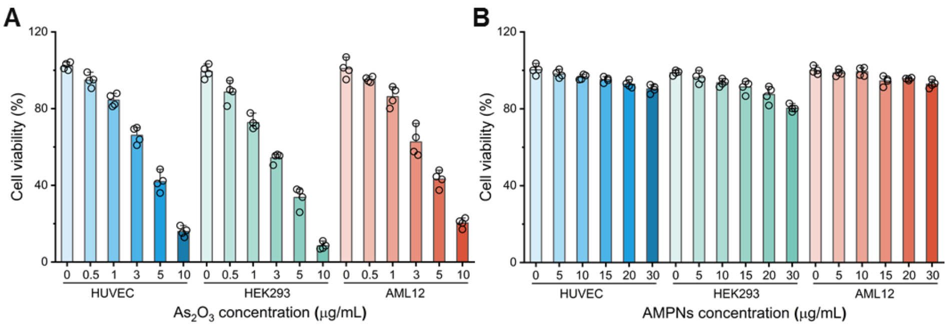

Figure S11. Relative cell viabilities of HUVEC, HEK293 and AML12 after co-incubation with (A) As_2O_3 and (B) AMPNs.

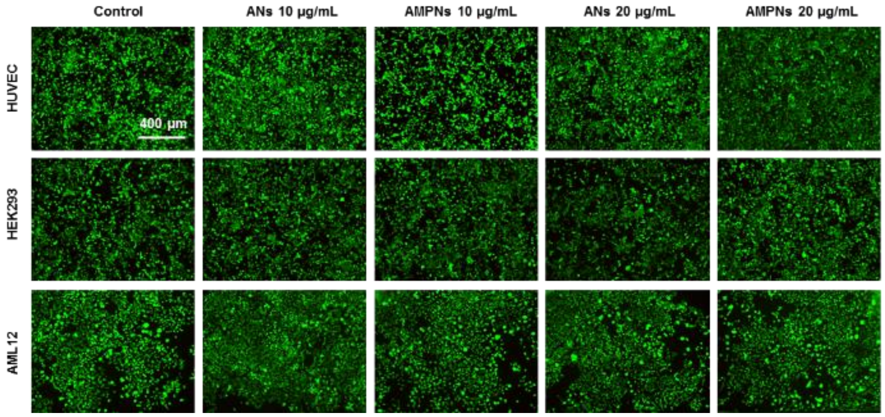

Figure S12. Live/dead stained of HUVEC, HEK293 and AML12 after treatments with ANs or AMPNs.

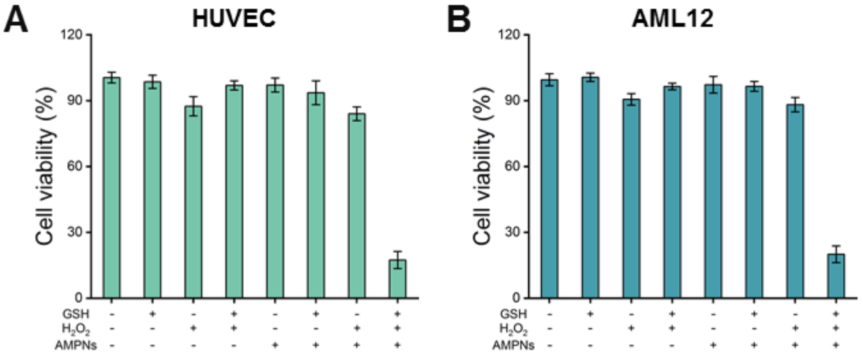

Figure S13. Relative cell viabilities of (A) HUVEC and (B) AML12 after various treatments.

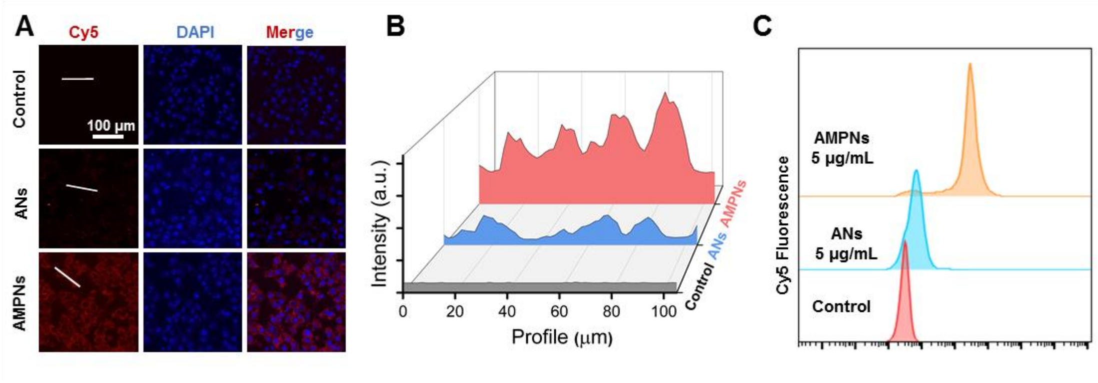

Figure S14. The (A) FC analysis, (B) CLSM images and (C) Fluorescence quantitative analysis of intracellular uptake after incubation with ANs or AMPNs for 4 h.

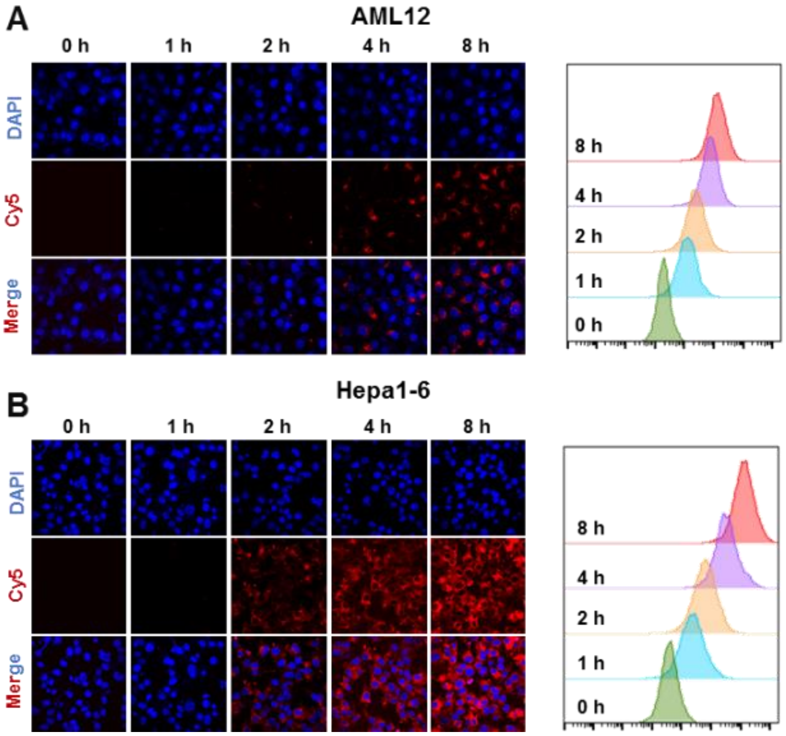

Figure S15. CLSM images and FC analysis of intracellular uptake after (A) AML12 and (B) Hepa1-6 incubation with AMPNs for different times.

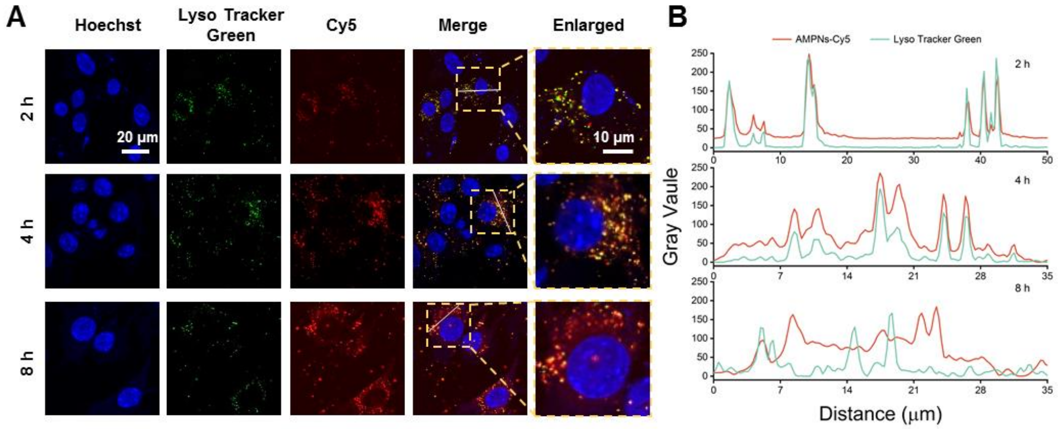

Figure S16. (A) CLSM images and (B) corresponding colocalization fluorescence intensity profiles of Hepa1-6 incubated with AMPNs for 2 h, 4 h or 8 h.

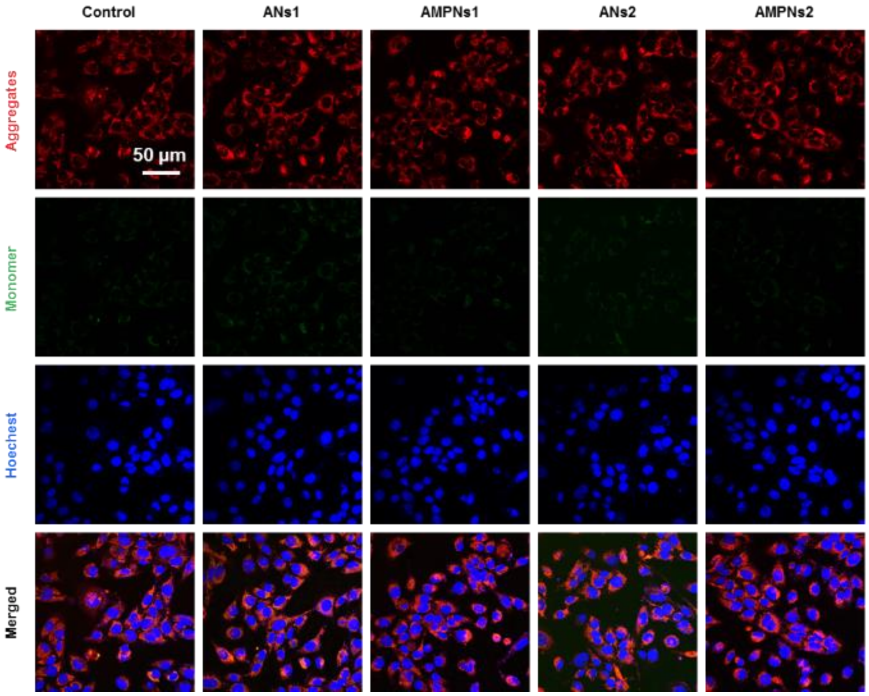

Figure S17. CLSM images of JC-1 staining after various treatments with HUVEC.

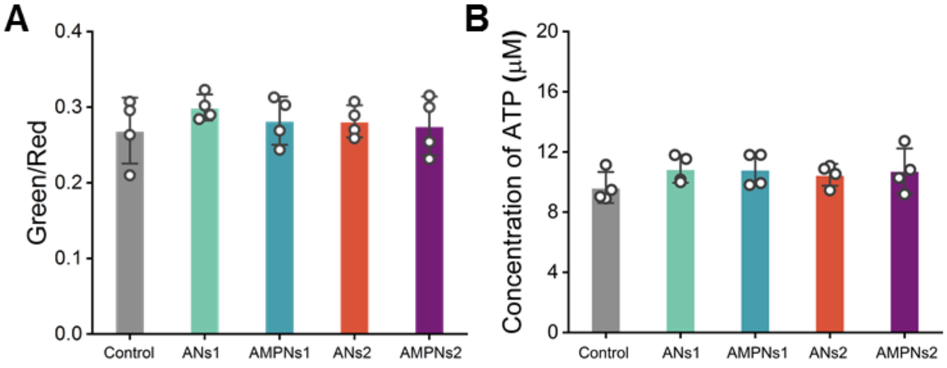

Figure S18. Quantification by (A) JC-1 stained and (B) ATP concentration after various treatments with HUVEC.

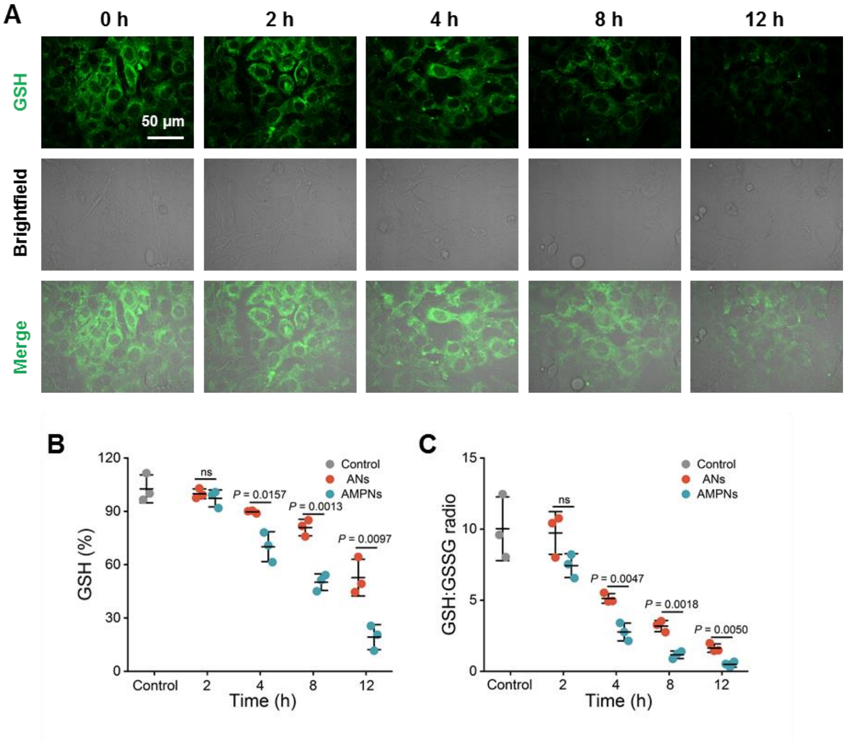

Figure S19. (A) CLSM images and (B), (C) quantitative analysis of GSH levels and the GSH:GSSG ratio after incubation with ANs or AMPNs for different times.

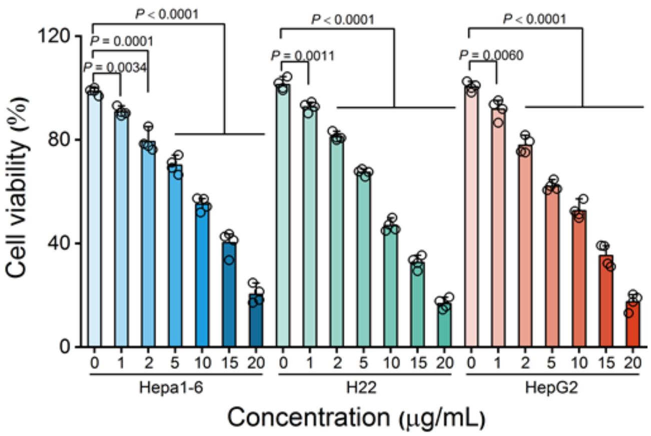

Figure S20. Relative cell viabilities of Hepa1-6, H22 and HepG2 cells after co-incubation with AMPNs.

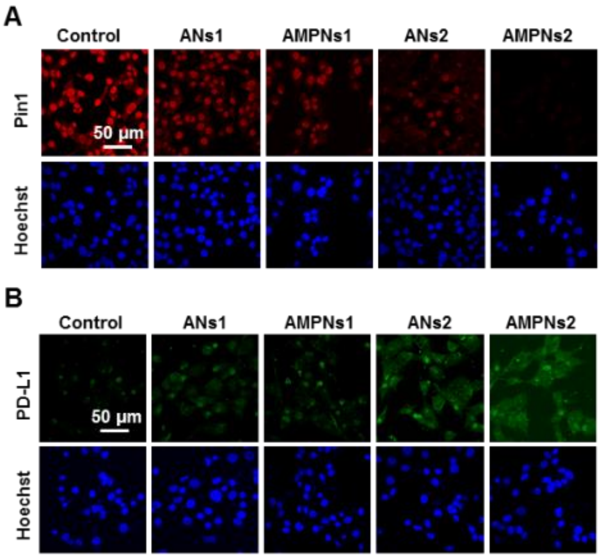

Figure S21. Immunofluorescence staining of (A) Pin1 (red) and (B) PD-L1 (green).

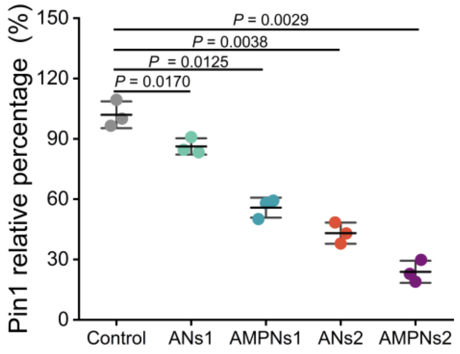

Figure S22. The relative percentage of Pin1 expression on Hepa1-6 cells after various treatment.

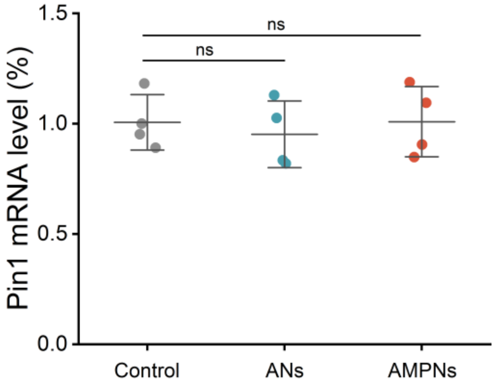

Figure S23. The relative mRNA level of Pin1 in Hepa1-6 cells following various treatments.

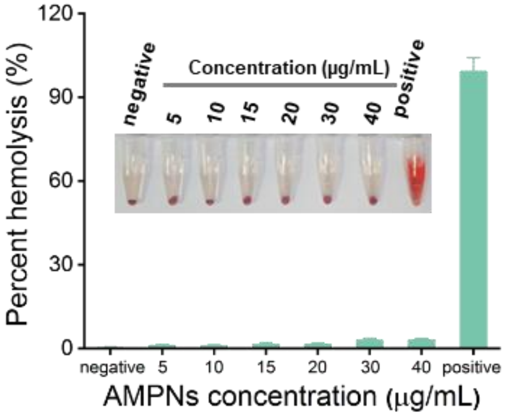

Figure S24. Hemolysis test of AMPNs.

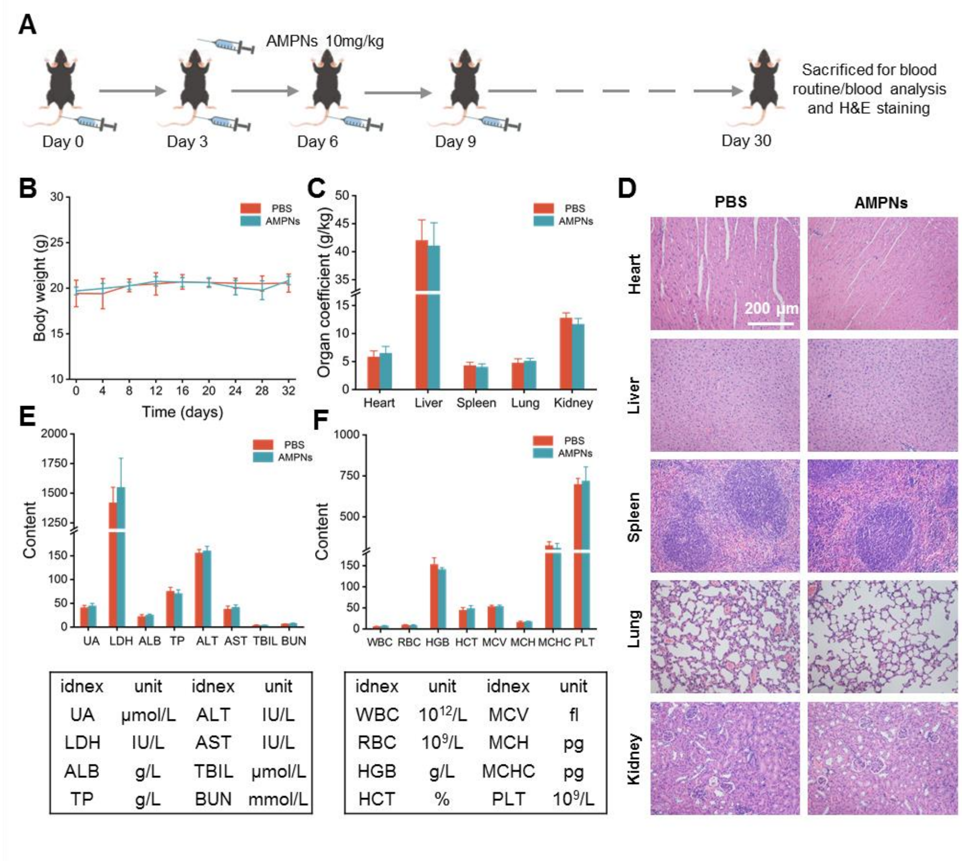

Figure S25. (A) Schematic illustration of the experimental design. (B) Body weight and (C) organ coefficient of mice treatment with PBS or AMPNs. (D) H&E staining images, (E) blood biochemistry and (F) blood routine index tests.

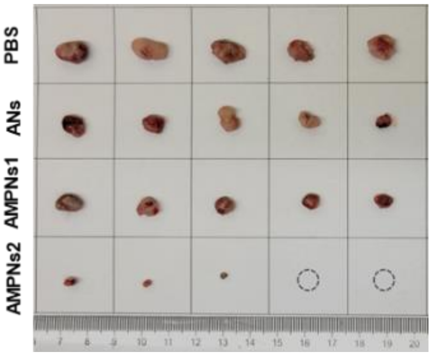

Figure S26. Tumor images of ANs- and AMPNs-treated mice.

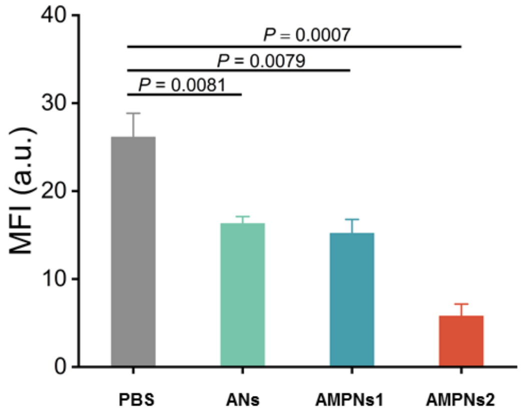

Figure S27. Quantitative analysis of Ki67 immunohistochemical staining

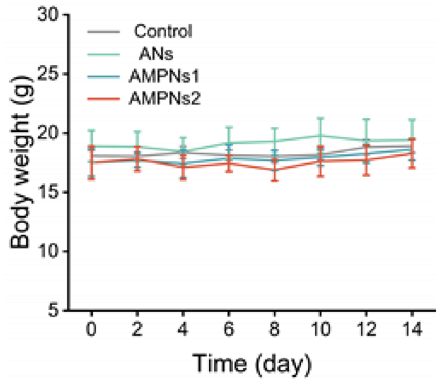

Figure S28. Body weight of mice over 14 days.

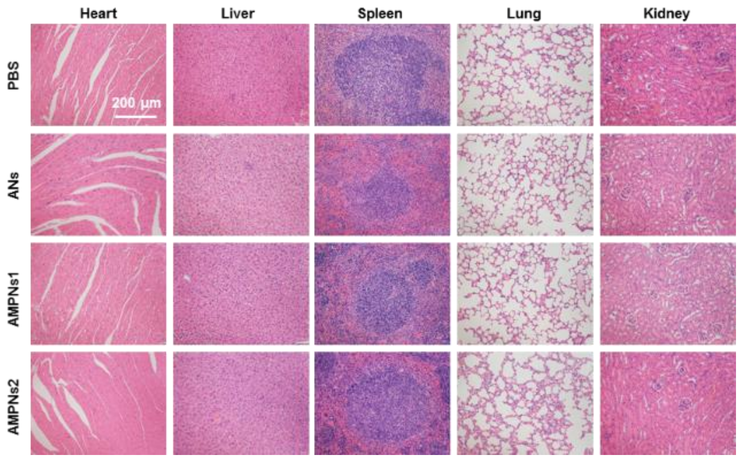

Figure S29. H&E staining of main organs of ANs- and AMPNs-treated mice.

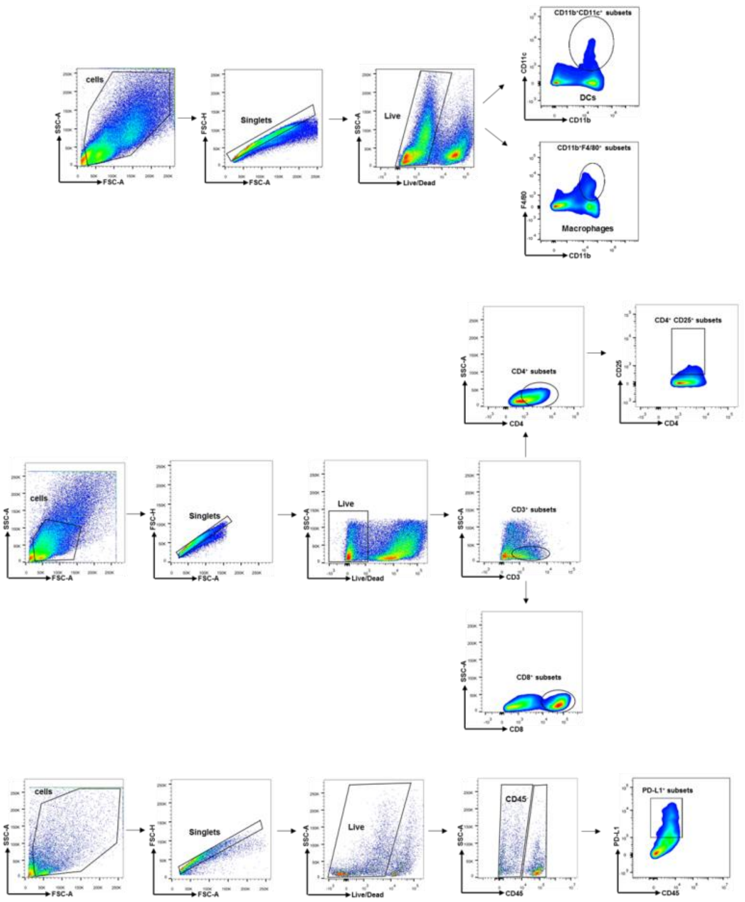

Figure S30. Gating strategy for flow cytometric analysis of leucocytes.

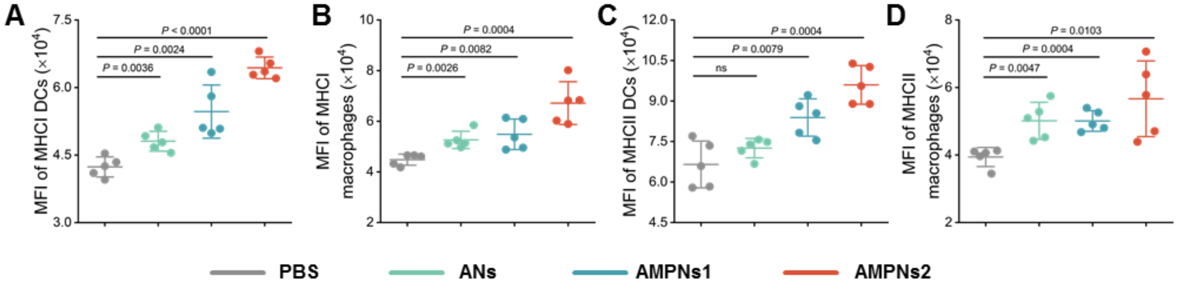

Figure S31. Aggregate graphs of MHCI in (A) DCs and (B) macrophages after treatment with or without ANs or AMPNs. Aggregate graphs of MHCII in (C) DCs and (D) macrophages after treatment with or without ANs or AMPNs.

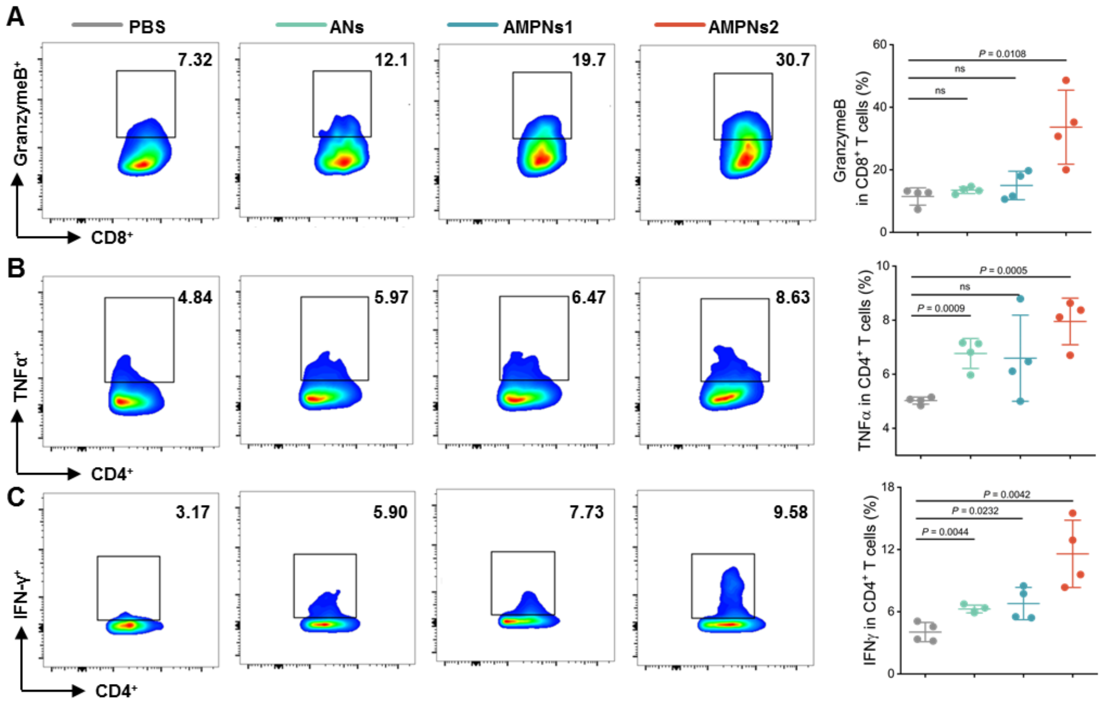

Figure S32. Representative FC analysis and aggregate graphs of (A) GranzymeB$^+$ CD8$^+$ T cells, (B) TNFα$^+$ CD4$^+$ T cells and (C) IFNγ$^+$ CD4$^+$ T cells.

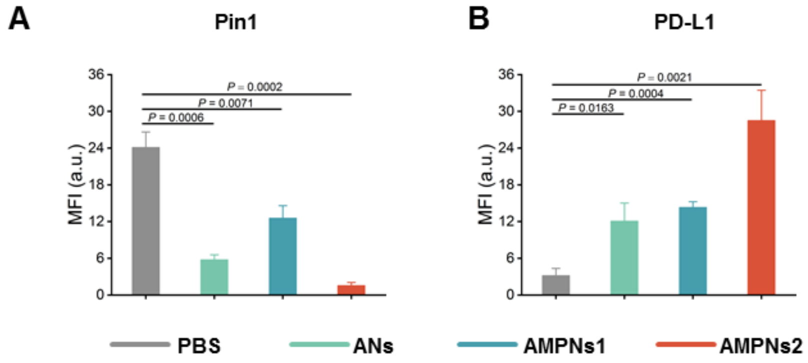

Figure S33. Representative FC analysis and aggregate graphs of (A) GranzymeB$^+$ CD8$^+$ T cells, (B) TNFα$^+$ CD4$^+$ T cells and (C) IFNγ$^+$ CD4$^+$ T cells.

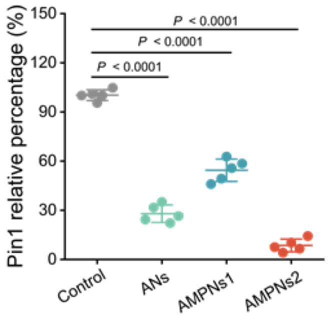

Figure S34. The relative percentage of Pin1 expression on tumor tissue after various treatment.

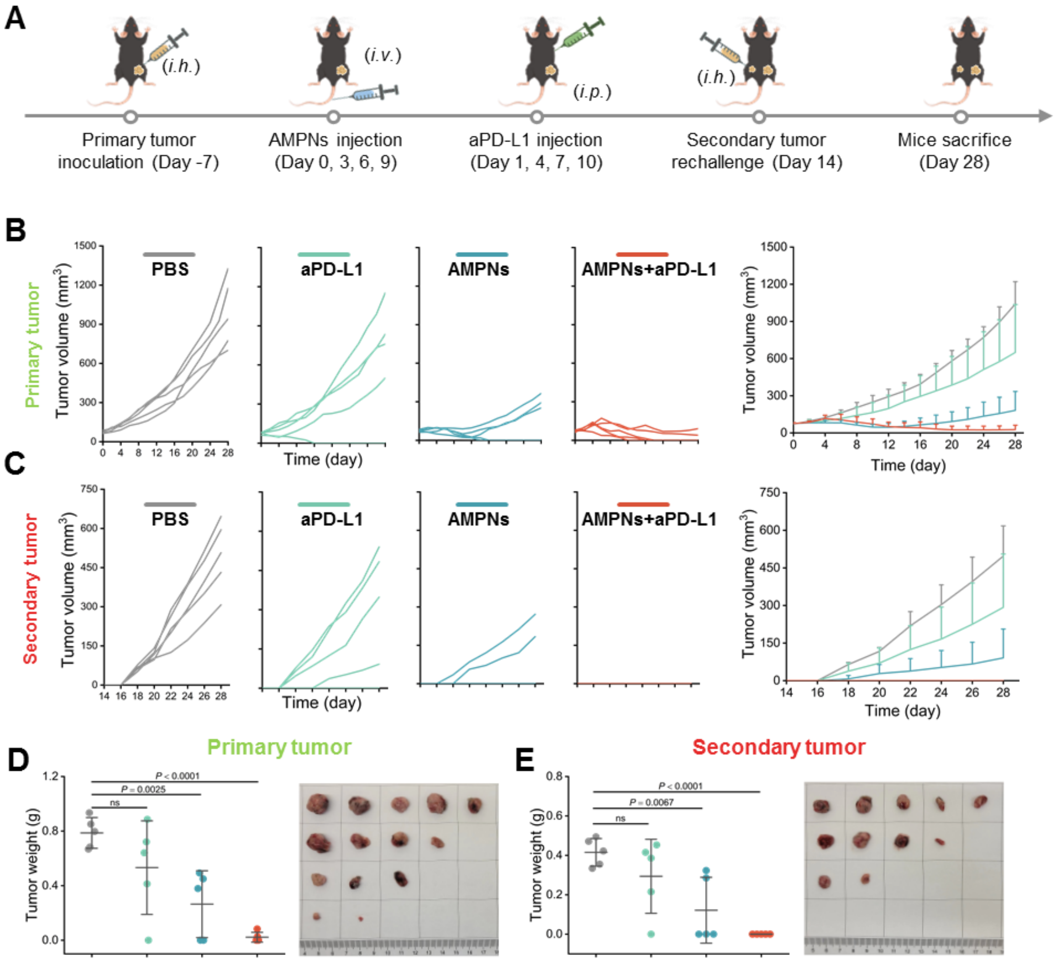

Figure S35. (A) Schematic of the experimental design. (B) Primary tumor and (C) secondary tumor growth curves. The tumor weight and images of (D) primary tumor and (E) secondary tumor.

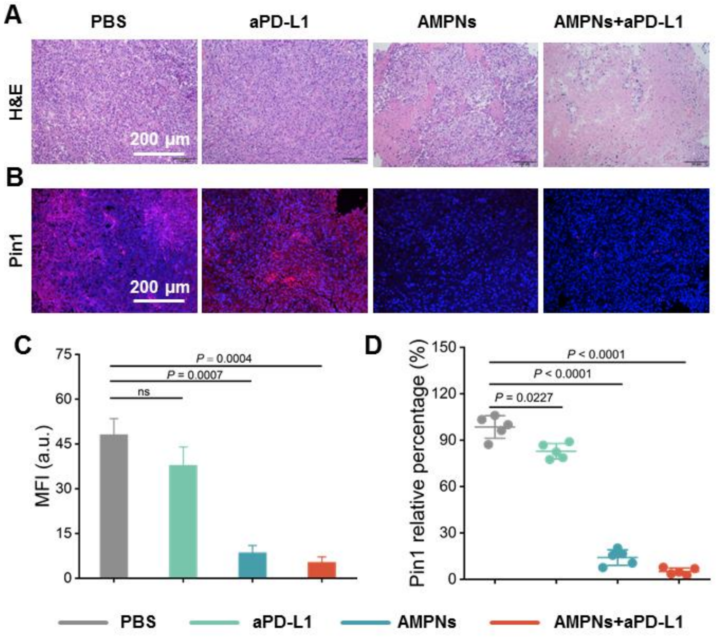

Figure S36. (A) H&E images of tumor tissue in different groups. (B) The immunofluorescence staining and (C) fluorescence quantitative analysis of Pin1. (D) The relative percentage of Pin1 expression on tumor tissue after various treatment.

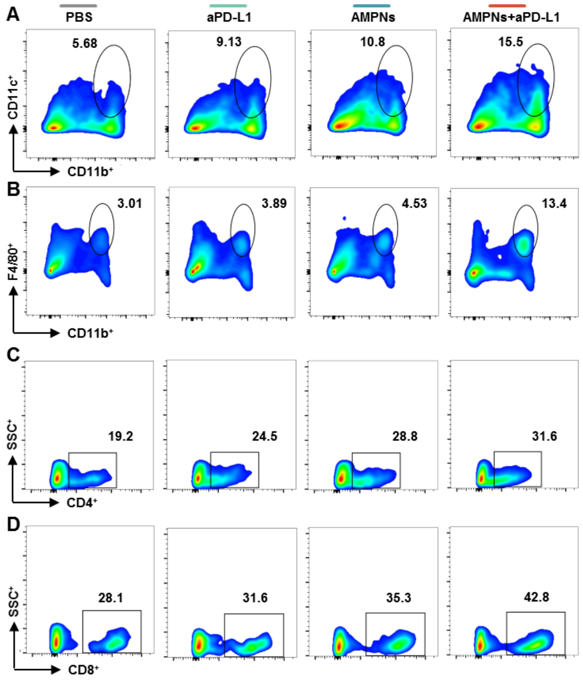

Figure S37. Representative FC analysis of (A) DCs, (B) macrophage cells, (C) CD4^{+} T cells, (D) CD8^{+} T cells in primary tumor.

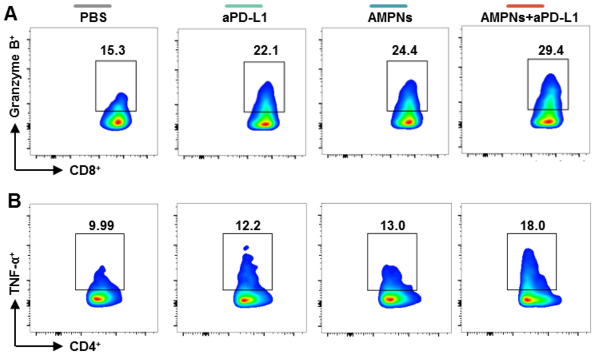

Figure S38. Representative FC analysis and corresponding quantification of (A) GranzymeB$^+$ CD8$^+$ T cells and (B) TNFα$^+$ CD4$^+$ T cells.

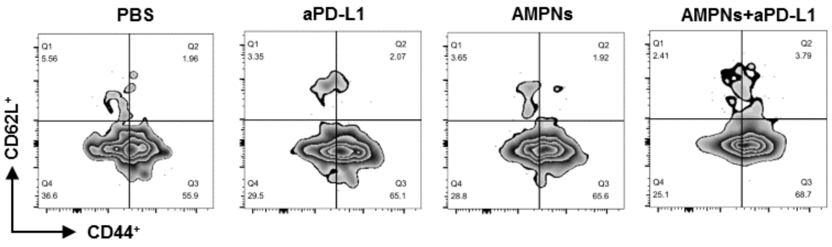

Figure S39. Representative FC analysis data for T_N (CD44$^-$ CD62L$^-$) and T_{CM} (CD44$^+$ CD62L$^+$) cells.

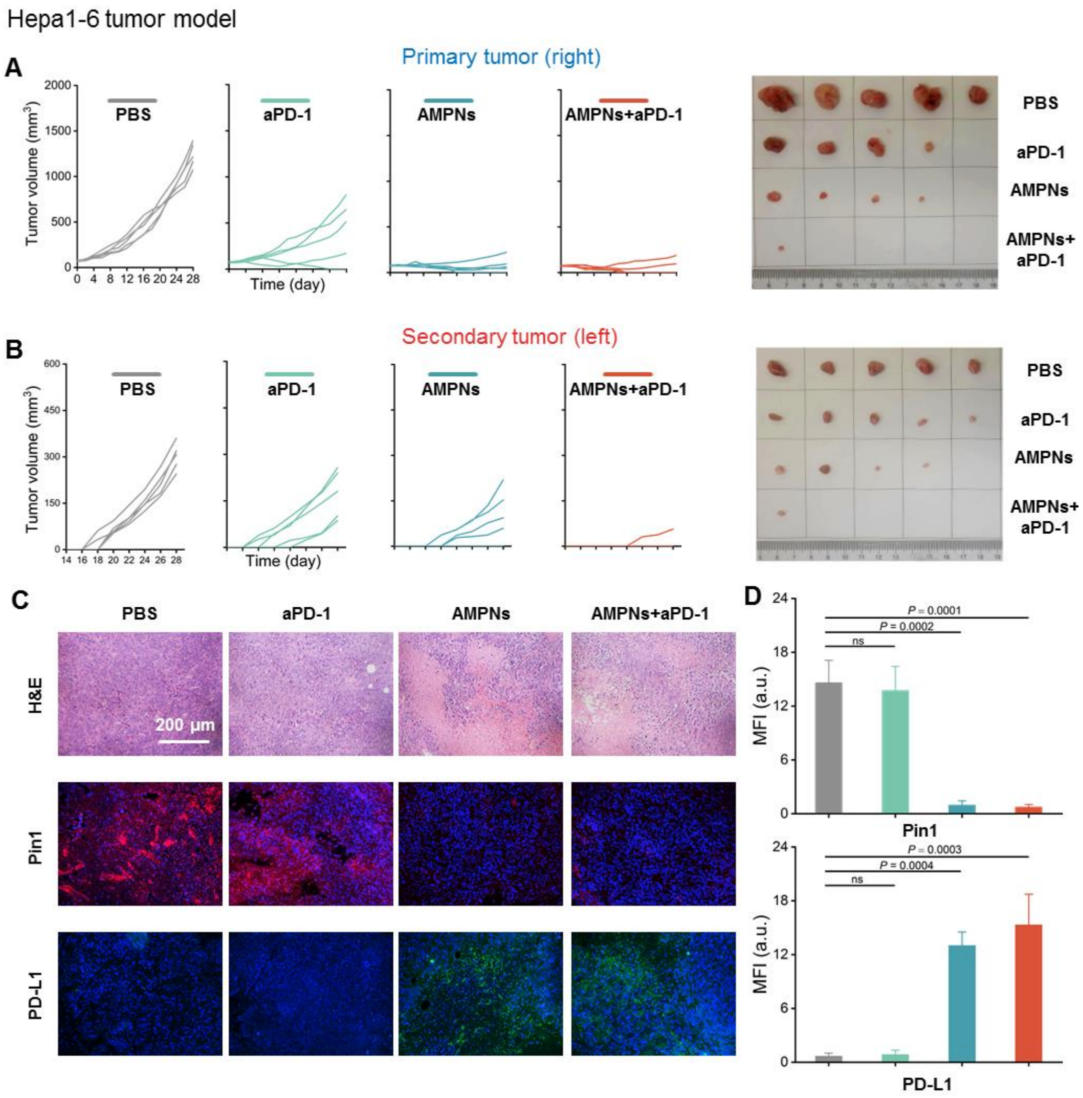

Figure S40. Tumor growth curves and tumor images for (A) primary and (B) secondary Hepa1-6 tumors after different treatments as indicated. (C) H&E, Pin1 and PD-L1 histology images for Hepa1-6 tumor tissue of different treated mice. (D) Fluorescence quantitative analysis of Pin1 and PD-L1.

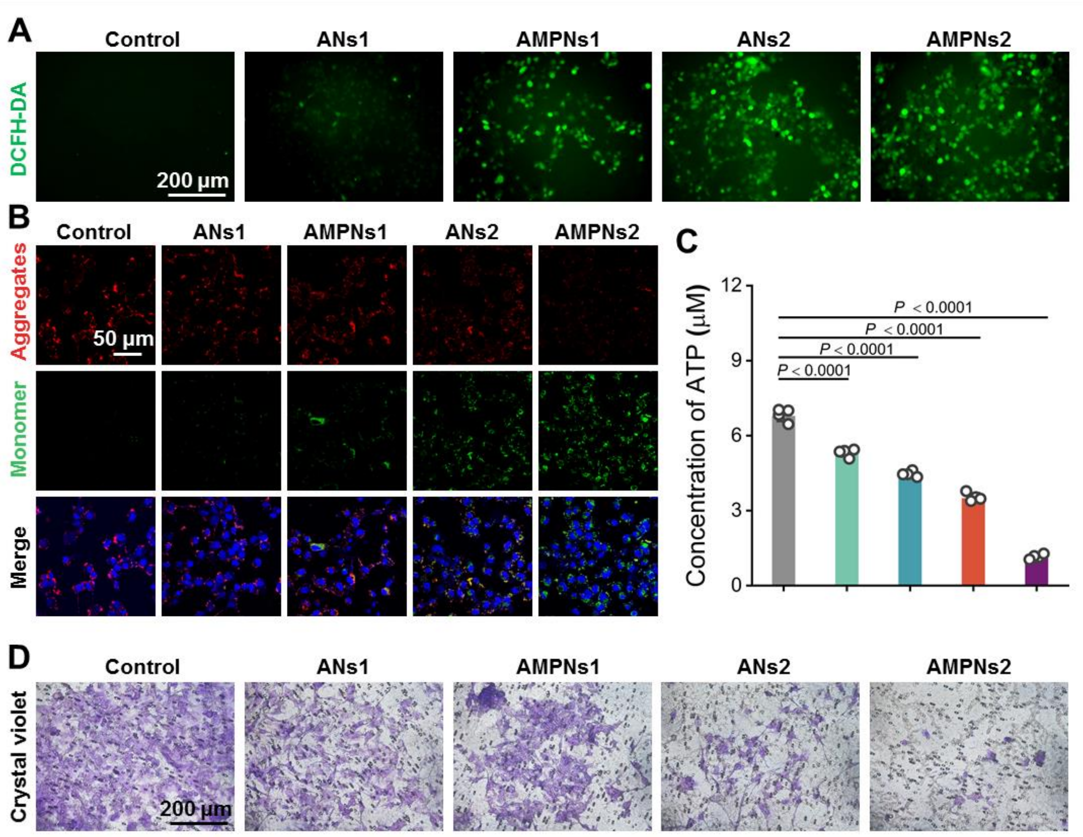

Figure S41. Alterations in (A) ROS levels, (B) mitochondrial depolarization, and (C) ATP concentration in 4T1 cells following treatment with ANs and AMPNs. (D) Representative images of 4T1 cells invasion after ANs and AMPNs treatments.

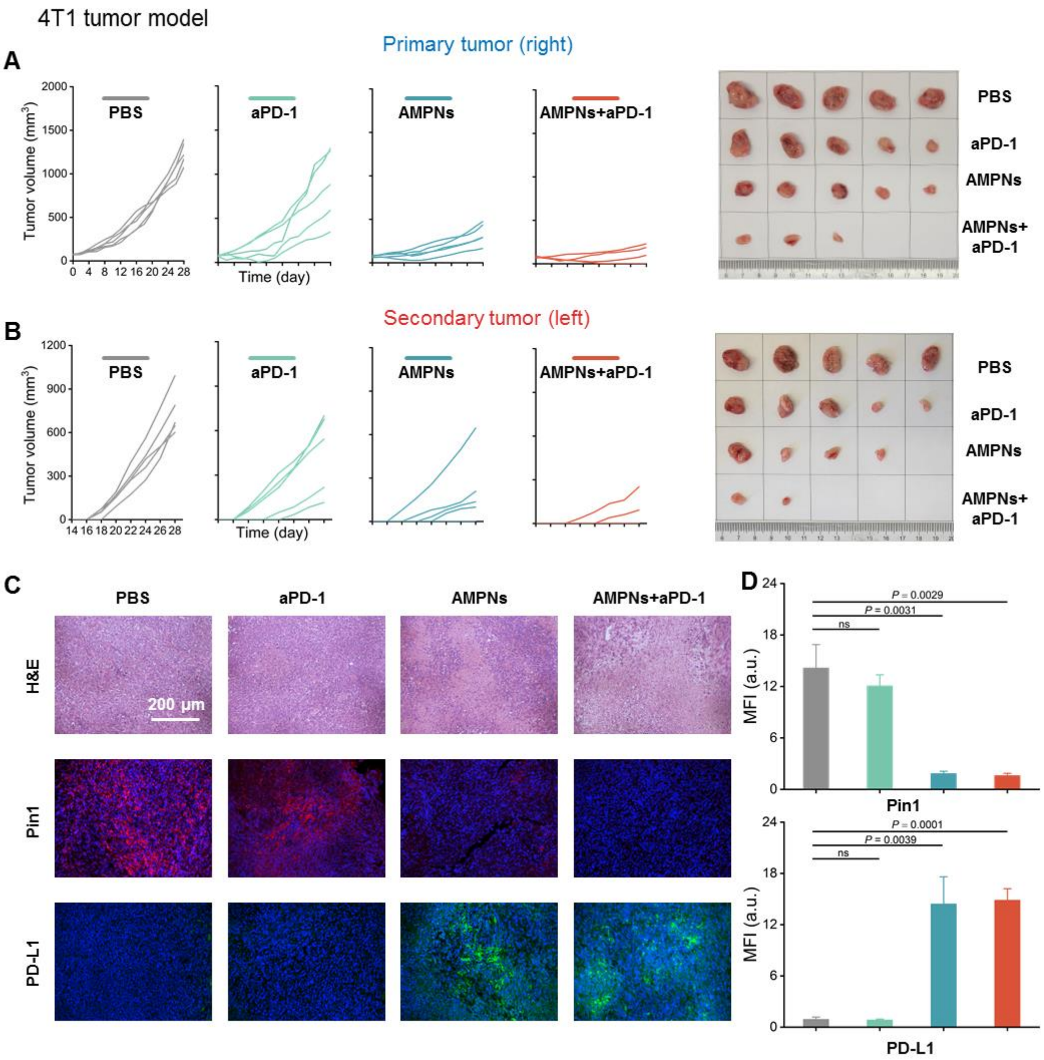

Figure S42. Tumor growth curves and tumor images for (A) primary and (B) secondary 4T1 tumors after different treatments as indicated. (C) H&E, Pin1 and PD-L1 histology images for 4T1 tumor tissue of different treated mice. (D) Fluorescence quantitative analysis of Pin1 and PD-L1.

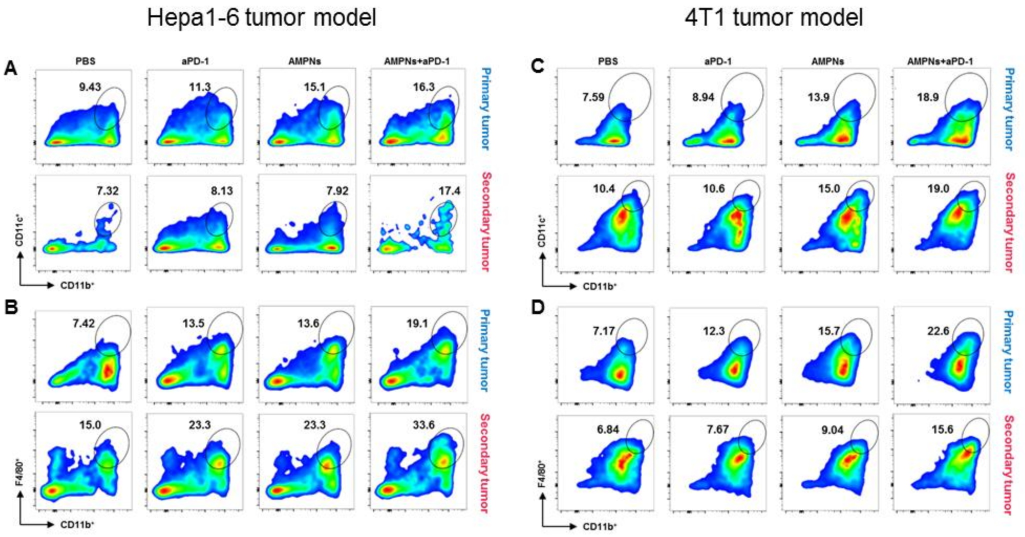

Figure S43. Representative FC analysis data for TME (A) DCs and (B) macrophages, with the primary and secondary Hepa1-6 tumor. Representative FC analysis data for TME (C) DCs and (D) macrophages, with the primary and secondary 4T1 tumor.

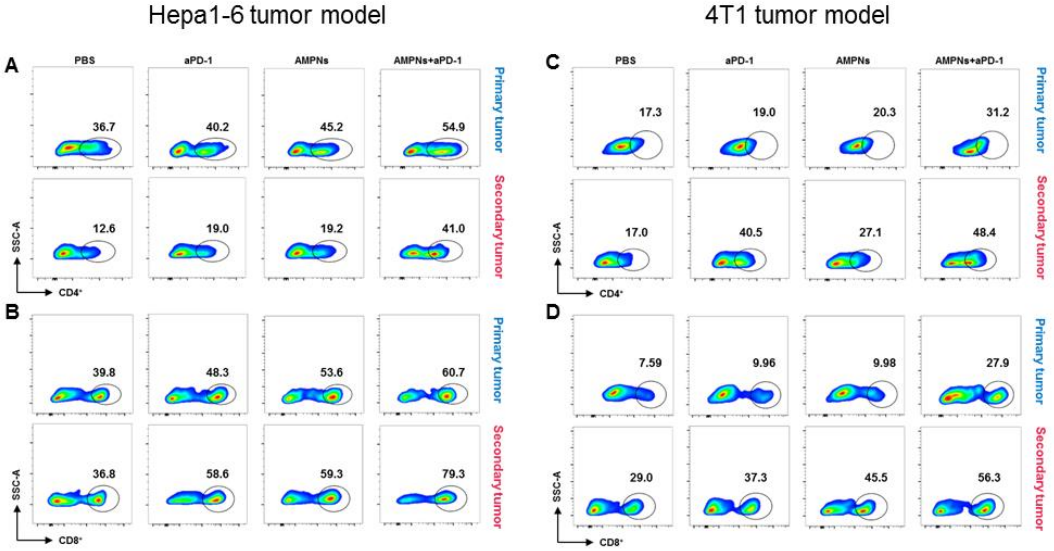

Figure S44. The FC results of (A) $CD4^+$ T cells and (B) $CD8^+$ T cells infiltration within the Hepa1-6 tumor after different treatments. The FC results of (C) $CD4^+$ T cells and (D) $CD8^+$ T cells infiltration within the 4T1 tumor after different treatments.

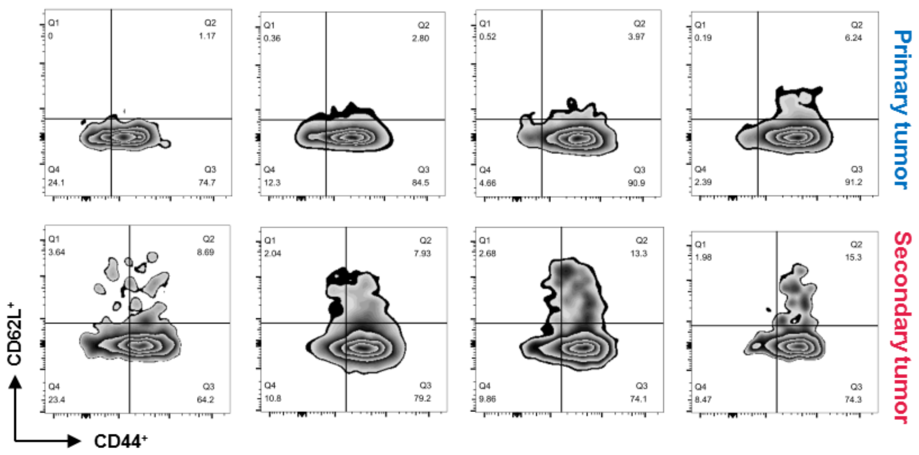

Figure S45. Representative FC analysis data for T_N ($CD44^- CD62L^-$) and T_{CM} ($CD44^+ CD62L^+$) cells with the primary and secondary tumor.

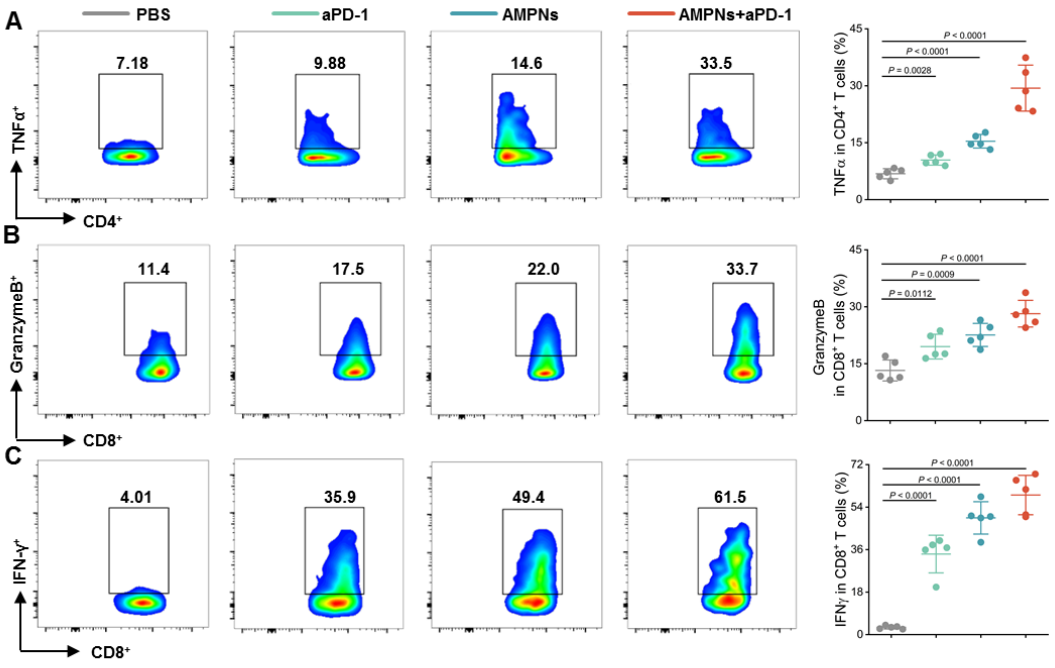

Figure S46. Representative FC analysis data and aggregate graphs of (A) $TNF\alpha^+$ $CD4^+$ T cells, (B) $GranzymeB^+$ $CD8^+$ T cells and (C) $IFN\gamma^+$ $CD8^+$ T cells.

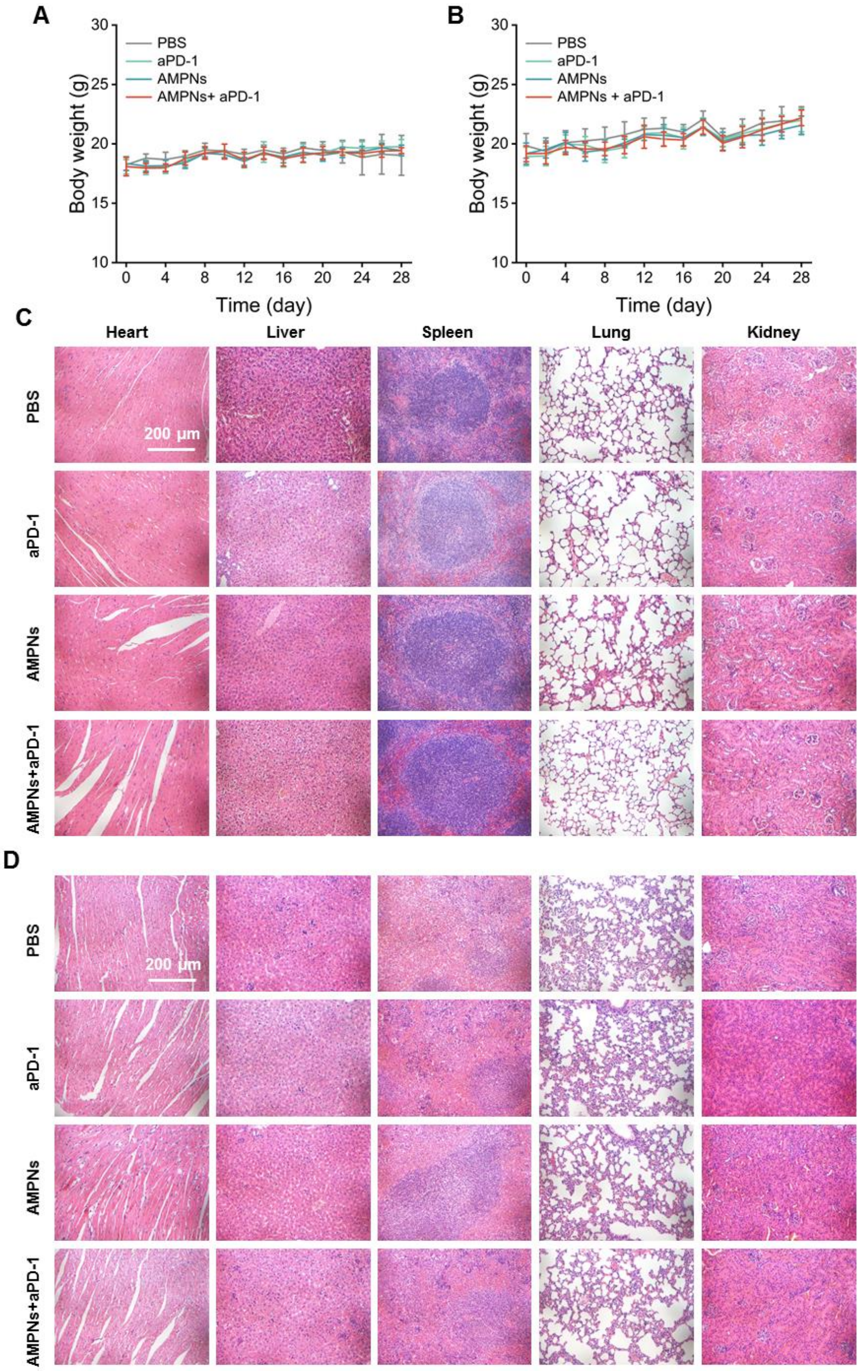

Figure S47. Body weight of (A) Hepa1-6 tumor-bearing mice and (B) 4T1 tumor-bearing mice over 28 days. H&E staining of main organs of (C) Hepa1-6 tumor-bearing mice and (D) 4T1 tumor-bearing mice in different groups.
